# Septic arthritis score (SAS) – a novel clinical prediction model for the probability of septic arthritis in the adult native knee

**DOI:** 10.1186/s12879-025-11306-6

**Published:** 2025-07-18

**Authors:** Jonas Tverring, Amelia Johansson, Omid Bornaei, Adam Lantz, Oskar Ljungquist

**Affiliations:** 1 Department of Clinical Sciences Helsingborg, Division of Infection Medicine, Lund University, Lund, Sweden; 2 Department of Orthopaedics, Helsingborg hospital, Helsingborg, Sweden; 3 Department of Infectious Diseases, Helsingborg hospital, Helsingborg, Sweden

**Keywords:** Septic arthritis clinical prediction model

## Abstract

**Background:**

Patients presenting with an acutely painful swollen joint represent a diagnostic challenge. We aimed to develop a clinical prediction model for septic arthritis (SA) in the adult native knee.

**Methods:**

We screened all synovial cultures in south Sweden in 2020 and 2021. We included cultures taken in the emergency department from adults’ native knees where SA was considered a differential diagnosis based on medical chart review. We developed a prediction model using logistic regression and performed internal validation using bootstrapping. We present a nomogram and an online calculator (http://sascore.org) for individual risk estimation, net benefit compared to usual care and treatment threshold recommendations.

**Results:**

A total of 668 patients were included from 2996 screened synovial cultures. The final septic arthritis score (SAS) included four variables: synovial-to-serum glucose quotient, synovial white blood cell count, abnormal synovial fluid appearance on visual inspection, and triage priority according to Rapid Emergency Triage and Treatment System (RETTS) vital signs. SAS had an optimism-adjusted area under the receiver operating characteristics curve of 0.971 (95% bootstrap confidence interval: 0.957 to 0.987). Clinicians provided empirical intravenous antibiotics to 47 out of 51 patients with a final diagnosis of SA and to 244 out of 617 patients without SA (92% sensitivity, 60% specificity). SAS had 92% sensitivity and 92% specificity at 10% probability for SA treatment threshold and 100% sensitivity and 79% specificity at 2% treatment threshold.

**Conclusion:**

The use of SAS would theoretically avoid 50–82% of unnecessary empirical antibiotics as compared to usual care in our cohort with retained or improved identification of actual septic arthritis of the native knee. External validation is warranted before clinical use.

**Supplementary Information:**

The online version contains supplementary material available at 10.1186/s12879-025-11306-6.

## Introduction

Patients presenting with an acutely painful and swollen peripheral joint represent a clinical challenge [1]. The differential diagnosis includes crystal-induced disease, osteoarthritis, traumatic injury, infection and a variety of systemic diseases [2]. Timely identification of septic arthritis (SA) is important. An infected joint left untreated can lead to cartilage damage [3, 4]. Even with contemporary care there is a high rate of poor functional outcomes [5]. Prompt adequate antimicrobial treatment decreases length of hospital stay and costs [6]. However, it is not desirable to admit and administer intravenous antibiotics to all patients with a single acutely painful joint. Most monoarthritis are not caused by an infection [7]. Over-admitting and overtreating individuals for septic arthritis risks leading to adverse events from potentially unnecessary antimicrobials in the individual [8], increased antimicrobial resistance in the population [9], increased costs and harm from a potentially avoidable hospital admission [10] and a possible delay in the initiation of directed therapy for the correct differential diagnosis.

The clinical challenge in the ED is to differentiate between septic and non-septic arthritis based on presenting symptoms and serum- and synovial markers before microbiology results are available. This challenge has been the focus of systematic [7] and narrative [11] reviews. Age, diabetes mellitus, rheumatoid arthritis, joint surgery, hip or knee prothesis, skin infection and human immunodeficiency virus infection are all established risk factors for developing a septic arthritis [1]. But that does not necessarily mean they are strong individual predictors in the average presenting patient [12]. Most authors agree that history, physical examination and serum test have little discriminatory value and that synovial white blood cell count (WBC) is the most useful predictor of SA [7, 13–15]. Even though recent reviews have provided positive and negative diagnostic likelihood ratios [1, 7] we still lack a reliable way to estimate the probability of septic arthritis in the individual patient based on a few readily available clinical and laboratory variables. The aim of the current study was to provide such an aid in clinical decision-making through the development and internal validation of a novel prediction model in adult patients presenting at the emergency department with an acutely painful and swollen native knee.

## Methods

### Study design, setting and population

This was an observational retrospective population-based cohort study based in Skåne in southern Sweden. Skåne has a population of 1.4 million people, and the Department of Clinical Microbiology in Lund is responsible for all microbiological diagnostics in the region. All cultures obtained from joints registered during the year 2020 and 2021 were screened for inclusion. Inclusion criteria were adults (≥ 18 years old) who had synovial fluid obtained for bacterial culture from a native knee at the emergency department (ED) where septic arthritis was considered a differential diagnosis (based on retrospective medical chart review). Exclusion criteria were samples obtained from joints other than knees, prosthetic knee joints and cultures obtained in other institutions than the ED or for a planned visit. Patients could only be included once in the study.

### Septic arthritis outcome definition

Septic arthritis was considered a positive outcome in this study with the purpose of developing a prediction model to answer the clinical question “Should this patient be treated with *Staphylococcus aureus*-active empirical intravenous antibiotics?”. We defined septic arthritis according to a modified version of Newman’s criteria [3], i.e., a patient with an acutely painful and swollen native knee joint where a synovial sample yielded a culture-proven clinically relevant bacterial pathogen. A deviation from this definition could be done if the medical chart review revealed that the culture had been taken through infected skin, *or* if the patient had received antibiotics prior to the culture being taken *and* two independent medical chart reviewers and the treating clinician all considered septic arthritis to be most likely diagnosis. We did not collect data on polymerase chain reaction (PCR) results from synovial fluid, so bacteria that do not grow on conventional cultures (e.g., *Borrelia burgdorferi*) were not identified as septic arthritis. *Neisseria gonorrhoea* in synovial or blood culture was not considered septic arthritis in the primary analysis but was considered in sensitivity analyses. Bacteria that belong to the microbiota of the skin, such as coagulase-negative staphylococci (CoNS, other than *Staphylococcus lugdunensis*) were not considered relevant organisms. eMethods in Supplementary file 1 contain a definition for false and true positives and negatives according to usual care.

### Participant and microbiology data

All medical records were reviewed by two study-trained final-year medical students according to a predefined case report form (see a list of variables in eMethods, Supplementary file 1). The medical records of patients deemed to have a positive outcome that did not receive antibiotics according to guideline and/or were not admitted to hospital were double-checked by an infectious disease specialist (OL and/or JT). Synovial fluids were cultured with direct streaking on agar plates. The BACTEC™ FX (BectonDickinson, Franklin Lakes, United States) blood culture system was used for blood cultures. Identification of bacteria was done using MALDI-TOF MS (Bruker Daltonics, Bremen, Germany). The study design was approved by the Swedish Ethical Review Authority (DNR-2021-05349-01). The need for informed consent was waived in this retrospective cohort study.

### Statistical analysis

Here is a summarized account of the statistical analysis. Please refer to eMethods in Supplementary file 1 for a more detailed description and Supplementary file 2 for the complete Stata code used in this paper. The sample size was event driven. We screened synovial fluid samples in 1-year intervals until we achieved ~ 10 events per the intended ~ 5 variables in a final prediction model. We considered 15 pre-specified variables as candidate predictors based on clinical experience, divided into primary, secondary and exploratory candidates. We chose the final variables in the model based on a trade-off between clinical experience and apparent predictive performance of the variables in univariate and multivariate logistic regression analyses. Potential candidates were investigated for type of missingness and replaced by multiple imputation if variables were considered missing at random and exceeding 5%. Variables were investigated for non-linearity and influential observations. The final prediction model was internally validated using bootstrap and evaluated using a calibration plot. We present area under the receiver operating characteristics curve (AUROC), sensitivity, specificity, positive predictive values (PPV), negative predictive values (NPV) and diagnostic likelihood ratios (LR+/-). We provide a nomogram for individual risk probability calculation and net benefit across treatment thresholds using decision curve analysis. Stata MP 18.0 was used for all statistical analyses.

## Results

### Patient characteristics

**Fig. 1:**
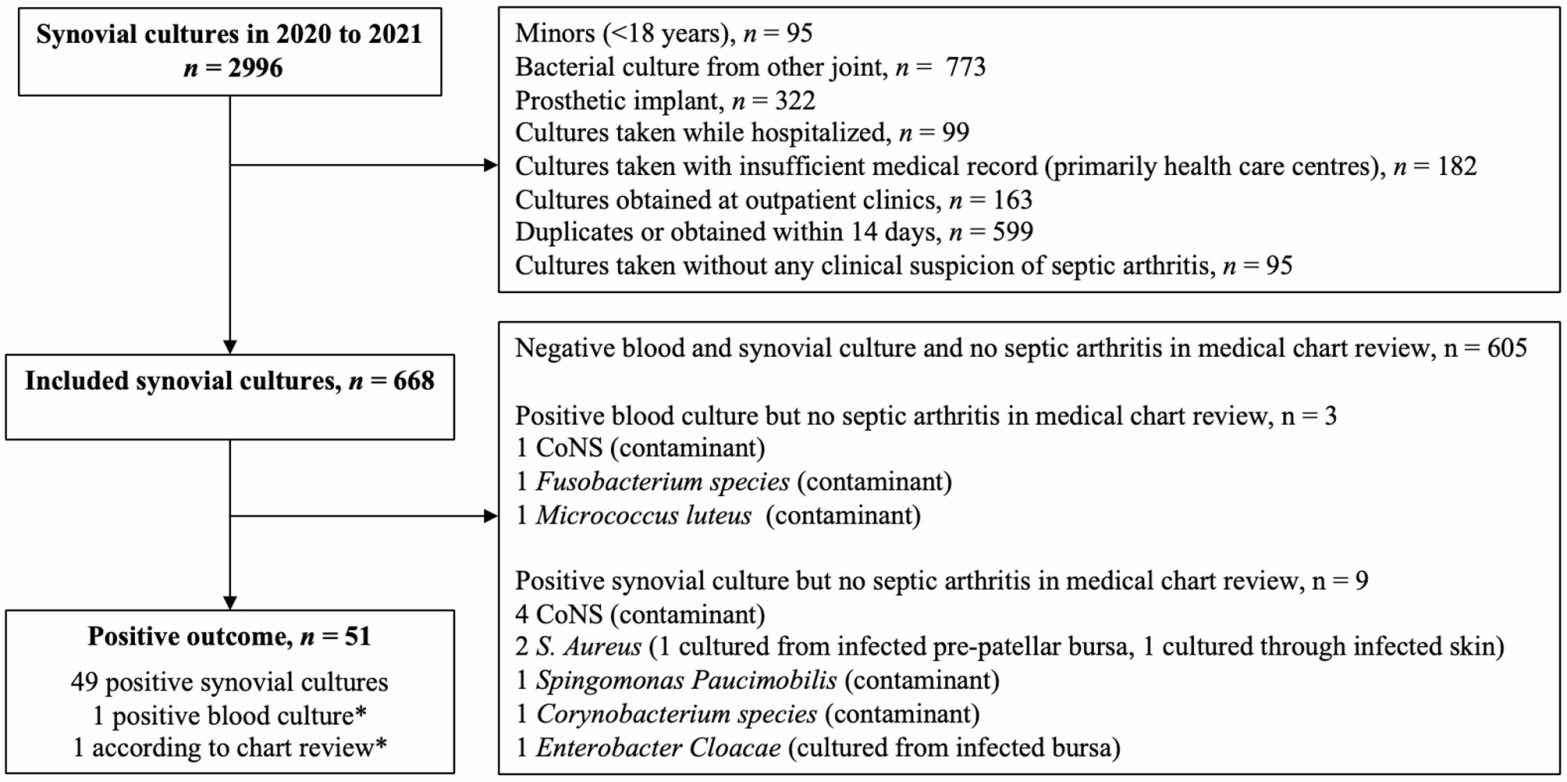
Flow chart of participants. Contaminants were defined according to a pre-specified list (see eTable S7). Cases excluded due to arthrocentesis going through infected skin or bursa were determined through medical chart. The two patients regarded as positive outcome without a positive synovial fluid culture were also regarded as positive outcome based on medical chart review by at least one final year medical student and one Infectious Diseases specialist. Out of these two patients, one had Streptococcus agalactiae in blood cultures and the other had received piperacillin/tazobactam prior to synovial and blood cultures

We screened a total of 2996 synovial bacterial cultures taken from joints during 2020 and 2021. Six-hundred-sixty-eight synovial cultures from 668 patients fulfilled the inclusion criteria (Fig. 1). Study participants were median 71 years old (range 18 to 97 years), 33% were female and had median Charlson comorbidity index (CCI) of 4 points (Interquartile range (IQR) 1 to 5 points), a median triage priority of 1 at ED (according to Rapid Emergency Triage and Treatment System, RETTS, where 3 is highest and 0 lowest) (eTable S1) and median C-reactive protein (CRP) of 81 mg/L (IQR 41 to 145 mg/L) (Table 1).

**Table 1:** Baseline characteristics of included patients

Variable	Septic arthritis (*n* = 51)	Non-septic arthritis (*n* = 617)	% missing
Age (years), median (IQR)	72 (61 to 83)	71 (55 to 81)	0
Female sex	27%	34%	0
BMI (kg/m^2^), median (IQR)	25 (23 to 29)	26 (24 to 30)	61%
Smoker (current)	14%	11%	1%
Symptom duration (days), median (IQR)	3 (1 to 5)	3 (2 to 6)	6%
Chronic kidney disease	31%	17%	0
Diabetes mellitus (any)	25%	17%	0
Rheumatoid arthritis	4%	12%	0
Charlson comorbidity index, median (IQR)	4 (2 to 6)	3 (1 to 5)	0
Triage priority (RETTS, 0 = lowest, 3 = highest), median (IQR)	2 (1 to 3)	1 (1 to 1)	14%
Temperature at ED (C°), median (IQR)	37.8 (36.9 to 38.6)	37.1 (36.7 to 37.6)	4%
C-reactive protein (mg/L), median (IQR)	188 (102 to 301)	75 (38 to 136)	5%
Serum WBC (10^9^/L), median (IQR)	10.8 (9.1 to 15.4)	10.2 (8.5 to 12.2)	21%
Length of hospital stay (days), median (IQR)	14 (9 to 21)	0 (0 to 5)	0

*BMI *Body Mass Index*, RETTS *Rapid Emergency Triage and Treatment System (based on vital signs),* ED *Emergency department,* WBC *White Blood Cell count

### Septic arthritis outcomes

Fifty-one (7.6%) out of 668 patients were considered to have a positive outcome. Relevant bacteria grew in 49 of these synovial cultures, with *Staphylococcus aureus* being the most common finding (50%, eTable S2). Two cases were considered septic arthritis based on medical chart review despite a negative synovial culture, where one had received antibiotics in the two weeks prior to the ED visit and grew *Streptococcus agalactiae* in the blood culture and the other had received piperacillin-tazobactam prior to obtaining cultures.

### Prediction model development and performance

The final prediction model, the septic arthritis score (SAS), included four variables: synovial-to-serum glucose quotient, synovial white blood cell count, abnormal synovial fluid appearance on visual inspection, and triage priority according to RETTS vital signs (see Table 2 for the full regression model). For a detailed account on the prediction model development, please refer to eResults, eFigure S1-S5 and eTable S3−4 in Supplementary file 1.

**Table 2:** Full final multivariable regression model for SAS

Variable	Z value	Coefficient (95% CI)	*p* value
_constant	−3.62	−3.82 (−5.90 to −1.75)	< 0.001
Synovial to serum glucose quotient *(Continuous variable from 0 to 1)*	−6.83	−7.03 (−9.06 to −5.02)	< 0.001
Triage priority according to RETTS *(4 categories from lowest 0 to highest 3*,* but entered as a continuous variable)*	4.81	1.72 (1.02 to 2.43)	< 0.001
Synovial white blood cell count *(Continuous variable from 0 to 250)*	3.02	0.02 (0.01 to 0.03)	0.003
Synovial to serum glucose quotient (Continuous variable from 0 to 1)	2.91	2.45 (0.80 to 4.10)	0.004

Number of obs = 668. Prob > chi2 < 0.001. Degrees of freedom = 4. Pseudo *R*^2^ = 0.61

*RETTS *Rapid Emergency Triage and Treatment System

**Fig. 2:**
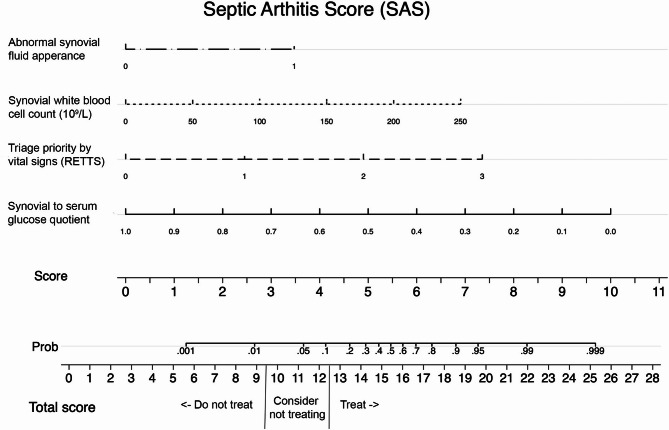
Nomogram for calculation of individual probability for a septic arthritis based on the four variables in the SAS prediction model. RETTS; Rapid Emergency Triage and Treatment System (based on vital signs)

SAS had a raw discriminatory ability based on AUROC of 0.974 and a raw model fit of 0.614 based on pseudo R^2^. Overfitting was estimated to 3.31%. On internal validation using 1000 replications bootstrapping, optimism-adjusted AUROC was 0.971 (95% bootstrap CI: 0.957 to 0.987) and Brier score (scaled) was 51.7%. SAS had a good calibration in the large with − 0.004 intercept (95% CI: −0.459 to 0.475) and an expected to observed ratio of 1.06 (95% CI: 0.829 to 2.144) with slight overestimation of risk probabilities based on slope 0.943 (95% CI: 0.716 to 1.180). See eFigure S6 for the calibration plot and bootstrap-adjusted model estimates in eTable S4. See eResults in Supplementary file 1 for results of sensitivity analyses. See eFigure S7-9 for Net Benefit (NB) of SAS compared to usual care, decision curve analysis and trade-off between false negatives and false positives across different SAS-based treatment thresholds. The probability for septic arthritis in the individual based on the four variables in the SAS model can be estimated using the nomogram in Fig. 2 (see eTable S5 for exact points). A draft online calculator for SAS can be found at http://sascore.org.

### Prediction model performance compared to usual care

**Fig. 3:**
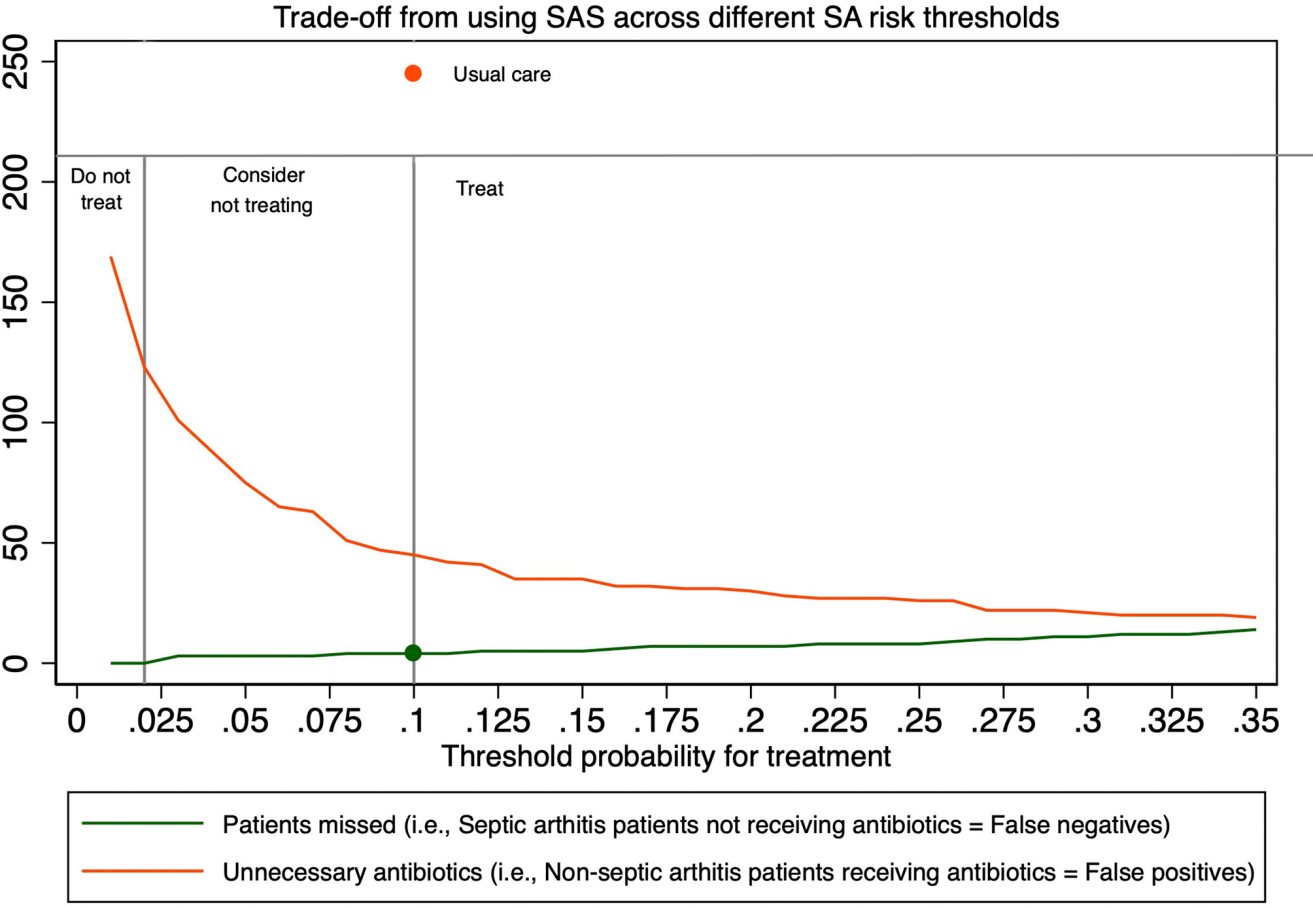
Graphical visualisation of the trade-off between patients with septic arthritis not receiving empirical antibiotics versus patients without septic arthritis receiving antibiotics when using the SAS prediction model across different treatment thresholds and the authors’ suggested cut-offs (2% and 10%). “Usual care” is defined by clinician’s choice to treat with empirical antibiotics in the cohort where 4 patients with septic arthritis did not receive intravenous antibiotics and 244 patients without septic arthritis received antibiotics (orange and green dot)

Clinicians administered intravenous antibiotics with activity against *S. aureus* to 47 out of 51 patients with septic arthritis and empirical antibiotics (oral or intravenous) to 244 out of 617 patients (40%) with a negative outcome (eTable S6). This corresponds to 92% sensitivity, 60% specificity, 63% accuracy and 5.7 patients treated with empirical antibiotics per positive outcome. The SAS model had 92% sensitivity and 92% specificity at a treatment threshold of 10% probability for septic arthritis. Using SAS at this threshold would theoretically avoid empirical antibiotics in 199 patients without septic arthritis (82% of those provided by clinicians) whilst failing to identify an equal number of patients with final septic arthritis as the clinicians in the cohort (4 out of 51). At a treatment threshold of 2% the SAS model had 100% sensitivity and 79% specificity. This would theoretically mean that we could provide empirical antibiotics to all patients with final septic arthritis in the cohort while still saving up to half of all unnecessary empirical antibiotics in patients without septic arthritis as compared to usual care (121 out of 244) (Table 3). The trade-off from using SAS across different treatment thresholds can be further appreciated in Fig. 3, where we have modelled the false negative rate (i.e., SA patients not receiving empirical antibiotics) versus false positive rate (i.e., patients receiving empirical antibiotics despite not having a final diagnosis of SA).

**Table 3:** Discriminatory performance of usual care versus the SAS model

	Nomogram points	Accuracy	False pos.	False neg.	Exchange rate	Sensitivity	Specificity	LR+	LR-	PPV	NPV
SAS at 10% TT	> 12.5	93%	45	4	1:1	92%	92%	12.6	0.09	52%	99%
SAS at 2% TT	< 9.5	81%	123	0	1:2.4	100%	79%	4.8	0	30%	100%
Usual care	N/A	63%	244	4	1:4.7	92%	60%	2.29	0.16	16%	99%

Usual care: is defined by medical chart review of the clinicians decision to give or not give empirical intravenous antibiotics to patients with septic arthritis or empirical oral or intravenous antibiotics to patients without septic arthritis

*TT *Treatment threshold, i.e., the SAS-derived probability at which treatment with empirical intravenous antibiotics is recommended. Exchange rate is the number of treatments provided compared to the number of true positives, *LR+/- *Positive diagnostic likelihood ratio*, LR- *Negative diagnostic likelihood ratio*, PPV *Positive predictive value*, NPV *Negative predictive value

### SAS treatment threshold recommendations

We suggest a stepwise clinical approach to treatment thresholds using the SAS model nomogram, dividing patients into three categories. Below 9.5 points, we recommend withholding empirical antibiotics (< 2% risk for septic arthritis and > 3 antibiotic doses per outcome positive patient). Between 9.5 and 12.5 points, we recommend considering withholding antibiotics and observing patients until culture results are obtained (2–10% risk for septic arthritis and 2–3 antibiotic doses per outcome positive patients). Above > 12.5 points, we recommend administering empirical *S. aureus*-active intravenous antibiotics (> 10% risk for septic arthritis and < 2 antibiotic doses or per outcome positive patient). The same thresholds and recommendations are also applied in the draft online calculator at http://sascore.org.

## Discussion

### Main findings

We have developed a novel septic arthritis score (SAS) prediction model that appears to enable a sizable reduction in unnecessary antibiotic administration while retaining a high level of correct identification of infection for adult patients presenting in the emergency department with a suspicion of septic arthritis in the native knee.

### SAS compared to other septic arthritis prediction models

We are not aware of any comparable prediction models for septic arthritis in adults. Kocher et al. presented a clinical prediction model for paediatric hip septic arthritis in 1999 based on four dichotomised variables (history of fever > 38.5 °C, non-weight bearing joint, erythrocyte sedimentation rate (ESR) > 40 mm/h and serum WBC > 12*10^9^/L) [16]. Compared to the Kocher model, we did not find fever to be particularly powerful predictor, serum WBC was very weak and while ESR was missing in most our patients, serum CRP (which was later added to the Kocher score as an ESR alternative at > 2 mg/dL) was only moderately predictive. The Kocher score did not include any predictor variables from synovial fluid which constitutes 75% of the SAS score. The prediction model proposed by Kocher et al. have since been evaluated in studies by the same [17] and other groups [18] with diminishing predictive performance and suggested adjustments to the included variables, cut-offs and even caution towards its use [19].

### Variables in SAS compared to findings in previous studies

Previous studies focusing on adult septic arthritis have not found strong predictive value from information on co-morbidities, physical examination, fever or serum markers such as CRP, WBC or ESR, but point to the recurrent prognostic performance of synovial WBC [1, 7]. While cut-offs between 25, 50 and 75 × 10^9^/L have been previously discussed [20, 21], our data shows that the probability for septic arthritis is linearly increasing with increasing synovial WBC (eFigure S4). This information should not be dichotomised [22]. Interestingly, synovial to serum glucose quotient proved a more powerful predictor than synovial WBC in the SAS model (eTable S4) which is not completely in line with previous studies [23, 24]. We acknowledge that the high performance of glucose quotient may represent overfitting to our data, and this added to our decision not to model glucose quotient cubically despite better performance (eFigure S3). To the best of our knowledge, abnormal synovial fluid appearance and triage priority according to vital signs have not been evaluated as a predictor of septic arthritis previously. We consider the four variables in the model taken together as clinically sound.

### Treatment thresholds for septic arthritis

Septic arthritis is widely considered the most serious differential diagnosis to an acutely painful swollen knee [1]. Overidentification and overtreatment of the condition could therefore be justified, i.e., a preference of sensitivity before specificity. In our cohort, clinicians were 92% sensitive (47/51) but were only 60% specific (1-244/617), administering antibiotics to 40% of patients without a final septic arthritis diagnosis. We argue that this represents unwarranted overtreatment, introducing a risk of adverse events from unnecessary antimicrobials, a potentially avoidable hospital admission, higher costs, and pressure for antimicrobial resistance. While a delay in therapy can cause damage to bone and cartilage, this was visible at first on day 3–8 in an experimental animal model [4] and in more than 1 week treatment delay in an observational study from the early antibiotic era [3]. Considering morbidity and mortality, septic arthritis is not equal to sepsis or septic shock. Native joint infections had around 3% attributable mortality in a recent cohort study [25]. Results from gram stains and/or cultures will be available the following day in most institutions, which gives room for a watchful waiting approach. We consider it reasonable to admit and administer intravenous antibiotics when there is at least 10% probability for septic arthritis. We acknowledge however that the treating clinicians will have information that was not considered or included in SAS, such as the general appearance of the patient, immunocompromise, or nuances in patient history, and that some clinicians will prefer a lower treatment threshold. We have therefore included a stepwise recommendation to consider withholding antibiotics when there is a 2–10% SAS-derived probability of septic arthritis.

### Strengths and limitations

One strength of this study was the population-based cohort study design that considered all synovial cultures in Skåne during two years. Another strength was the use of contemporary statistical methodology for prediction model development [26–28] and a consideration of the clinical applicability. A third strength is that 3 out of 4 included variables in the prediction model are reliably available from medical records and not subjective. The study is primarily limited by its retrospective design. Data collection was done by final year medical students and not all entries were double-checked by a physician specialist. There were considerable missingness in key variables that had to be imputed. We also acknowledge that some variables will have been less reliable in medical chart review compared to a prospective design (e.g., synovial fluid appearance). We also acknowledge that the triage priority system (RETTS) used in the model is not internationally widespread and that information from a more recognised risk stratification score would have been preferred (e.g. NEWS-2). We did not include results for non-culture-based microbiology analyses (e.g. 16 S PCR) on synovial fluid which means that we will not have identified some pathogens (e.g., *Borrelia burgdorferi*), we did not include septic arthritis cases where synovial cultures were not sent, and we excluded repeated episodes in the same patient. We also did not consider percentage Polymorphonuclear neutrophils (PMNs) or lactate in synovial fluid as potential predictors.

### How to use SAS and the next step

We acknowledge that the herein developed Septic Arthritis Score (SAS) has not been externally validated, and we do not recommend clinicians to use the prediction score before the model’s prognostic performance has been repeated in other settings. We plan to externally validate the model in the near future, but we also strongly encourage other groups to investigate SAS. We expect that the apparent performance will be lowered in external validation but that it will still be useful for an improved risk stratification compared to usual care. Even if the score is validated, its calculated points and the suggest cut-offs should be used with caution when approaching a patient. It should not replace clinical judgement but function as an aid in clinical decision making. It is not meant to be used in other joints than the knee, in children, in heavily immunocompromised individuals, in patients with septic shock or in patients with prosthetic joints. and. For those who want to try the SAS model for the sake of interest, a draft online calculator can be found at http://sascore.org.

## Conclusion

The herein developed SAS prediction model seems to offer highly accurate probability classification for patients with suspected septic arthritis of the native knee. This could potentially contribute to an avoidance of unnecessary empirical antibiotics in patients with an acutely painful swollen knee. External validation is warranted before clinical use.

## Supplementary Information

Supplementary Material 1.

Supplementary Material 2.

Supplementary Material 3.

## Data Availability

Data can be received from the corresponding author on reasonable request.

## Abbreviations

AUROC: Area Under the Receiver Operating characteristics Curve
CCI: Charlson Comorbidity Index
CI: Confidence Interval
CoNS: Coagulase-Negative Staphylococci
CRP: C-reactive protein
ED: Emergency Fepartment
ESR: Erythrocyte Sedimentation Rate
RETTS: Rapid Emergency Triage and Treatment System
LR: Diagnostic Likelihood Ratios
NB: Net Benefit
NPV: Negative Predictive Values
PCR: Polymerase Chain Reaction
PMN: Polymorphonuclear Neutrophils
PPV: Positive Predictive Values
SA: Septic Arthritis
SAS: Septic Arthritis Score
WBC: White Blood cell Count
