# Supplementary material for: Septic arthritis score (SAS) – a novel clinical prediction model for the probability of septic arthritis in the adult native knee
Source: BMC Infect Dis. 2025 Jul 18;25:926. doi: 10.1186/s12879-025-11306-6 (PMC12275251; doi:10.1186/s12879-025-11306-6)
Supplement: Supplementary file 1 — Supplementary Material 1. [file 12879_2025_11306_MOESM1_ESM.docx]

Supplementary File 1

SAS – A novel clinical prediction model for the probability of septic arthritis in the adult knee

*Tverring J, Johansson A, Bornai O, Lantz A and Ljungquist O*

**Index**

eMethods 1 p. 1-3

eTable S1-S2 p. 4

eResults 2 p. 5-6

eFigure S1-S5 p. 7-9

eTable S3 p. 10

eTable S4 p. 11

eFigure S6 p. 11

eTable S5-6 p. 12-13

eFigure S7-8 p. 14-15

eTable S7 p. 15-18

eReferences p. 19

**eMethods**

*Clinicians’ identification definition*

We considered the clinician to have made a correct identification of septic arthritis in the ED if the patient received *S. aureus*-active intravenous antibiotic (true positive). Patients who were treated with oral or intravenous antibiotics despite a negative outcome were regarded as being overtreated (false positive). Patients not receiving antibiotics and not experiencing the outcome were regarded as true negatives and patients not receiving *S. aureus*-active intravenous antibiotic in the ED despite having a positive outcome were regarded as false negatives (even if they received oral antibiotics).

*Methods - prediction model variable selection*

The prediction model candidates were pre-specified and chosen to represent different and comprehensive aspects of the clinical situation based on clinical experience and former known association with the outcome (1, 2). The candidates that were included in the final model represent a trade-off between the pre-specified priority of candidate predictors and the apparent predictive performance in the data at hand. We considered a total of 15 variables to be potential predictors of septic arthritis in the emergency department (ED). Five variables were considered primary candidates: synovial WBC, synovial-to-serum glucose quotient, normal versus abnormal synovial fluid on visual inspection, serum C-reactive protein and redness of skin surrounding the knee joint. Synovial fluid appearance was initially collected as 6 categories from the medical chart review: A) normal looking synovial, B) synovial fluid with debris, C) Synovial fluid with pus, D) synovial fluid with clear blood, E) synovial fluid with blood and debris, F) synovial fluid with blood and pus. We simplified this into a dichotomized variable where “normal synovial fluid” corresponded to A and D, i.e., normal looking synovial fluid with or without blood but not pus or debris, and all else considered abnormal appearance. We considered three secondary candidates: Triage priority based on vital signs in ED (Rapid Emergency Triage and Treatment System (RETTS), from lowest 0 to highest 3, (3), ear temperature at ED and serum white blood count. We lastly considered 7 exploratory candidates: Symptom duration, age, sex, Charlson comorbidity index (CCI), range of motion in the knee, previous rheumatoid arthritis diagnosis and diabetes mellitus. All variables were analysed univariately towards the outcome on their original scale using logistic regression. Primary variables with a very strong association towards the outcome (pseudo R^2^ > 20%) directly qualified to be part of the final prediction model. Primary and secondary variables with a strong association towards the (pseudo R^2^ > 10%) were added to the model if they individually added significant value to the primary variables in a Chi^2^ likelihood ratio test (LR test). Secondary and exploratory variables with a moderate association towards the outcome (pseudo R^2^ > 5%) were added to the model if they individually added significant value to the primary and secondary variables in the model in a LR test.

*Statistical analysis*

After the predictors in the final model were chosen, we investigated the variables for missing data and type of missingness. We performed multiple imputations using chained equations if any of the variables included in the model had missingness at random above 5% and performed multiple imputation diagnosis numerically and graphically. All predictor variables in the model were then individually investigated for a non-linear association towards the outcome using logistic regression. We investigated the graphical relationship between 4-categorical, cubical and 4-knot restricted cubic spline compared to linear modelling and whether non-linear modelling improved predictive performance in a LR test. We searched for collinearity and influential observations in the final prediction model and screened for a limited number of clinically plausible interactions. We estimated the raw predictive performance of the prediction model using the area under the receiver operation characteristics curve (AUROC) for discrimination and pseudo R^2^ for model fit. We performed interval validation of the model using 1000 replication bootstrap and investigated the performance graphically using a calibration plot and numerically by estimating the expected:observed outcomes, calibration in the large, calibration slope and optimism-corrected AUROC with 95% confidence interval (CI). We presented a nomogram for calculating the individual probability for SA based on the variables in the prediction model. We calculated the Net Benefit (NB, defined as NB = true positive rate – [false positive rate * exchange rate])) across different treatment thresholds (i.e., probability for SA) and presented them graphically using Decision Curve Analysis (DCA). We also compared the apparent performance of the clinician’s ability to make a correct identification of septic arthitis in the ED versus the performance of the developed prediction model across treatment thresholds. We performed sensitivity analyses regarding complete case analyses, exclusion of influential observations, exclusion of patients receiving antibiotics 2 weeks prior to study entry and by adjusting the outcome definition in relation to synovial and blood culture results and medical chart review.

**eTable S1.** Rapid Emergency Triage and Treatment System (RETTS) vital signs. RETTS is used in triage to assign suitable waiting times before the first assessment by a physician.

|  | 3, Red | 2, Orange | 1, Yellow | 0, Green |
| --- | --- | --- | --- | --- |
| A | Blocked airway or stridor |  |  |  |
| B | Respiratory rate >30 or <8  SaO_2_<90 with oxygen (O_2_) | Respiratory rate >25  SaO_2_<90 without O_2_ | SaO_2_≤95 | SaO_2_>95 without O_2_ |
| C | Heart rate >130 if sinus rythm, else >150  SBP <90 | Heart rate >120 or <40 | Heart rate >110 or <50 | Heart rate 50-110 |
| D | Unconscious or cramps | Somnolence | Acute disorientation | Alert |
| E |  | Temperature >41˚or <35˚ | Temperature >38.5˚ |  |

**eTable S2.** Microbiological findings in synovial and blood cultures

| **Bacterial species** | **Synovial culture** | **Blood culture** |
| --- | --- | --- |
| *S. aureus* | 25 | 13 |
| *S. dysgalactiae* | 8 | 4 |
| *S. agalactiae* | 4 | 2 |
| *E. coli* | 4 | 3 |
| *E. faecalis* | 3 | 1 |
| *E. cloacae* | 2 | 1 |
| *S. capitis* | 2 |  |
| Corynebacterium species | 1 |  |
| *H. influenzae* | 1 | 1 |
| *P. aeruginosa* | 1 |  |
| *S. caprae* | 1 |  |
| *S. epidermidis* | 1 | 1 |
| *S. paucimobilis* | 1 |  |
| *S. mitis* | 1 | 1 |
| *S. pneumoniae* | 1 |  |
| *S. sanguinis* | 1 | 1 |
| *S. aureus + S. constellatus* | 1 | 1 |
| Fusobacterium species |  | 1 |
| *M. luteus* |  | 1 |
| Total | 58 (8.7%) | 31 (4.6%) |

**eResults**

*Prediction model development*

Synovial-to-serum glucose quotient (glcq) and synovial white blood cell count (swbc) were strong individual primary predictors for septic arthritis at pseudo R^2^ of 0.38 and 0.27, respectively, and qualified directly to the final prediction model. Abnormal synovial fluid appearance by visual inspection (sabnormal), serum C-reactive protein (CRP) and triage priority (by RETTS vital signs, retts) were all individually moderately strong outcome predictors with a pseudo R^2^ of 0.19, 0.16 and 0.12, respectively **(eTable S3)**. On multivariable analysis synovial fluid appearance and triage priority added significant value to the model but CRP did not (*p*=0.20). There was considerable missingness in the four variables included in the final prediction model; 29% for glcq, 12% for swbc, 14% for retts and 8% for sabnormal. These missing values were imputed using multiple chained equations and non-missing patient variables (*n*_var_ = 312). Diagnostic plots showed good distribution of imputed variables (**eFigure S1-S2**). The continuous and categorical variables in the model were investigated for a non-linear relationship towards the outcome (**eFigure S3-S5**). Retts and swbc were both linearly related to the outcome. Glcq revealed a graphical and numerical advantage for cubic modelling (LR test *p*=0.0001 and pseudo R^2^ 0.44 vs 0.48, **eFigure S3**) indicating that the probability for SA remained high at quotients near 0 and decreased more steeply at quotients near 1 as compared to linear modelling. We still chose to model glcq linearly in the final model because we prioritized ease of use in clinical practice in favour of a slight increase in predictive performance. We found no signs of collinearity, no significant interactions in clinically relevant variables (sabnormal and swbc, swbc and retts, swbc and glcq, respectively) and 10 potential influential observations (based on -0.2<DFbeta>0.2). The logistic regression parameters for the full multivariable prediction model can be found in **Table S4**.

*Results – Net benefit*

The Net Benefit (4) of usual compared to the SAS model across different treatment thresholds or exchange rates can be visualised in a decision curve analysis (DCA) graph in **eFigure S7 (**see overfit corrected DCA in **eFigure S8**). The DCA graph shows that the SAS model outperforms both a “treat all”-strategy and usual care performance across all treatment thresholds.

*Sensitivity analyses*

The model fit and discriminatory were robust in sensitivity analyses using a complete case analysis (pseudo R^2^ 0.574, AUROC 0.966, n=373), when excluding patients who received antibiotics in the two weeks prior to recruitment (pseudo R^2^ 0.58, AUROC 0.972, n=578) and when excluding cases with influential observations (pseudo R^2^ 0.70, AUROC 0.987, n=659), respectively. The SAS model performance was lower when ignoring the chart review and considering all positive synovial cultures as septic arthritis except contaminants (Pseudo R^2^ 0.522 and AUROC 0.950, n_SA_=52/668) and even lower when also considering traditional contaminants as septic arthritis (Pseudo R^2^ 0.425 and AUROC 0.905, n_SA_=58/668).

**
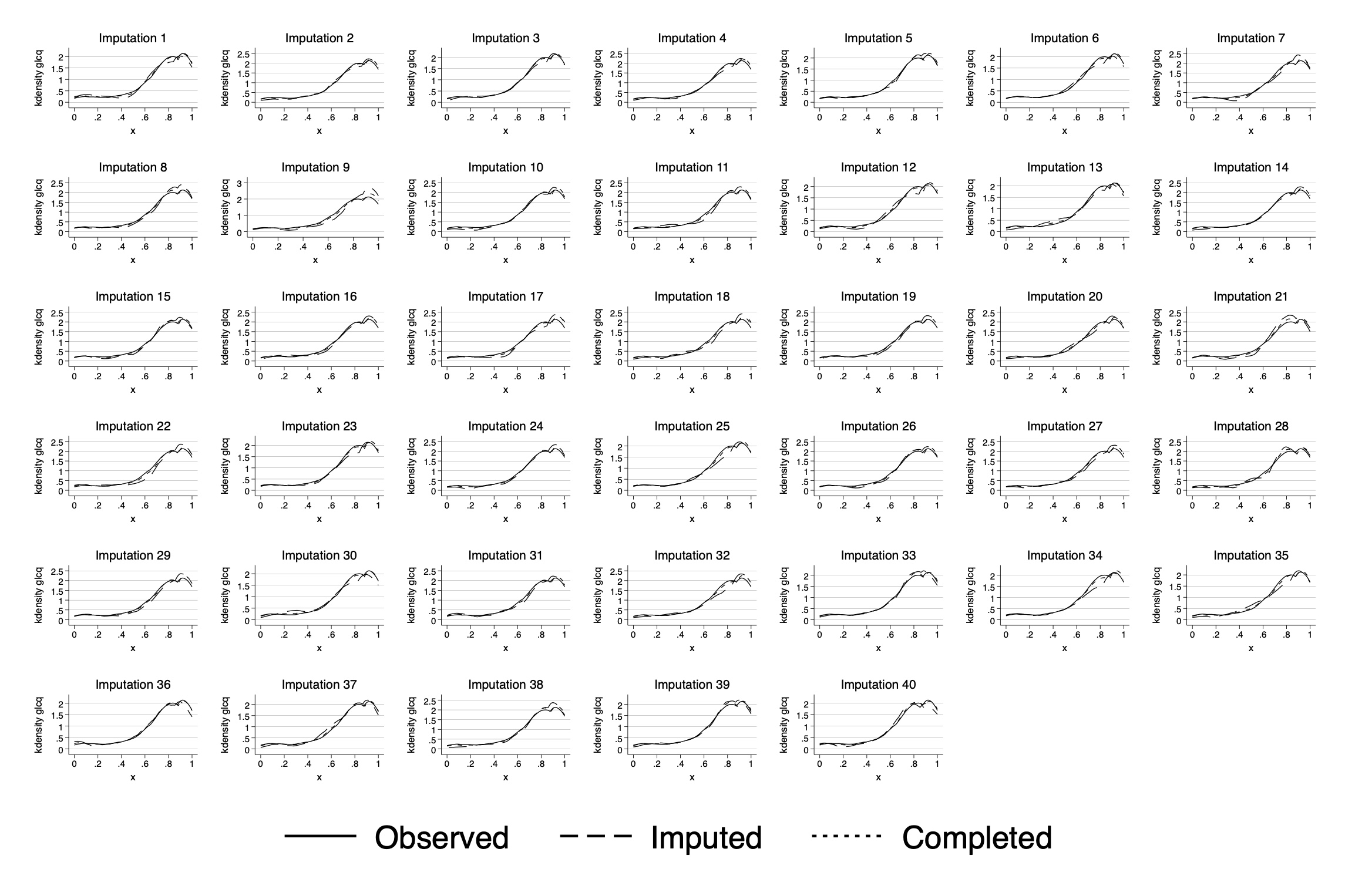
eFigure S1.** Distribution of imputed synovial to glucose quotient variables across 40 datasets.

**
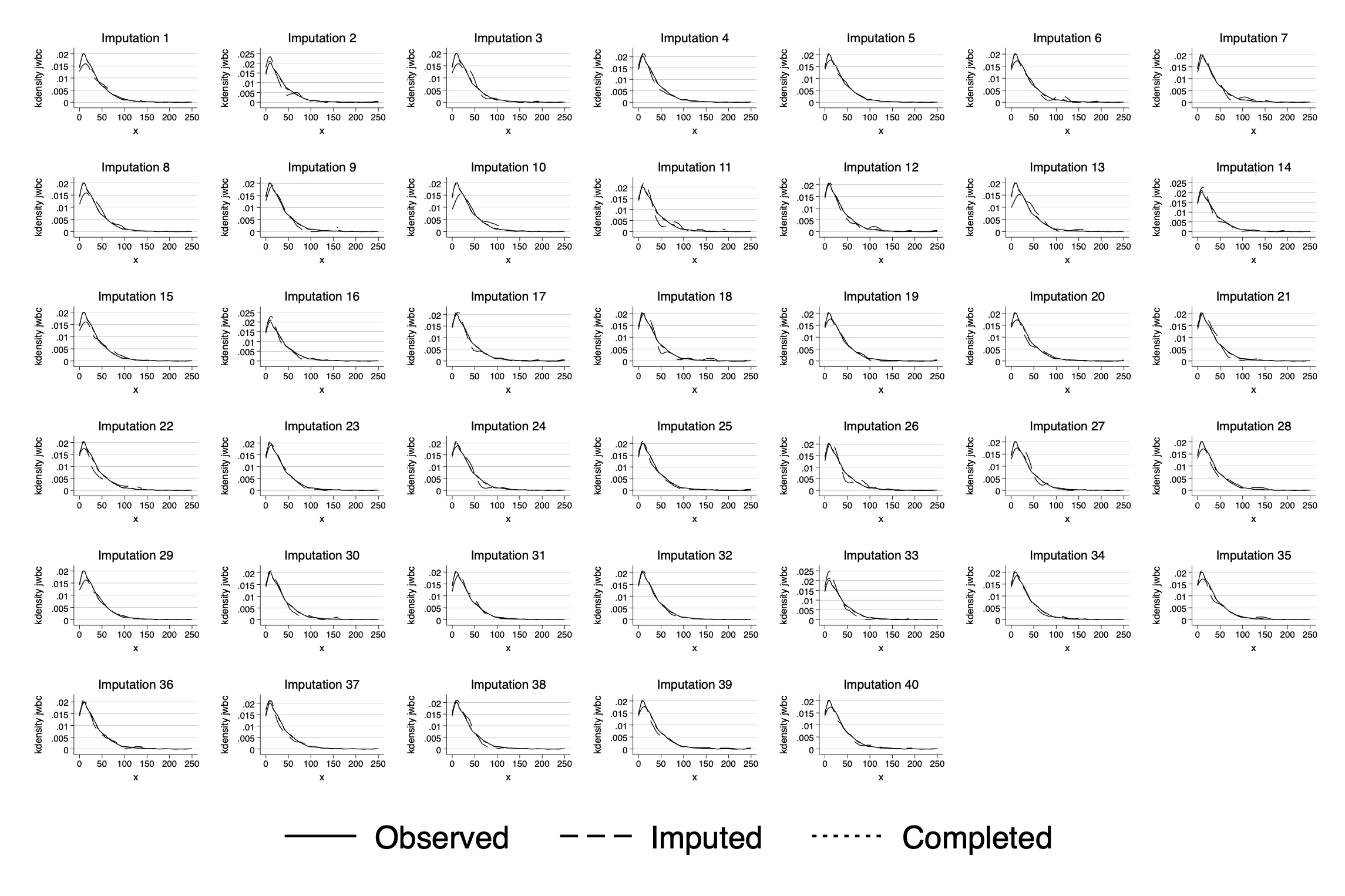
eFigure S2.** Distribution of imputed synovial white blood cell count variables across 40 datasets.

**
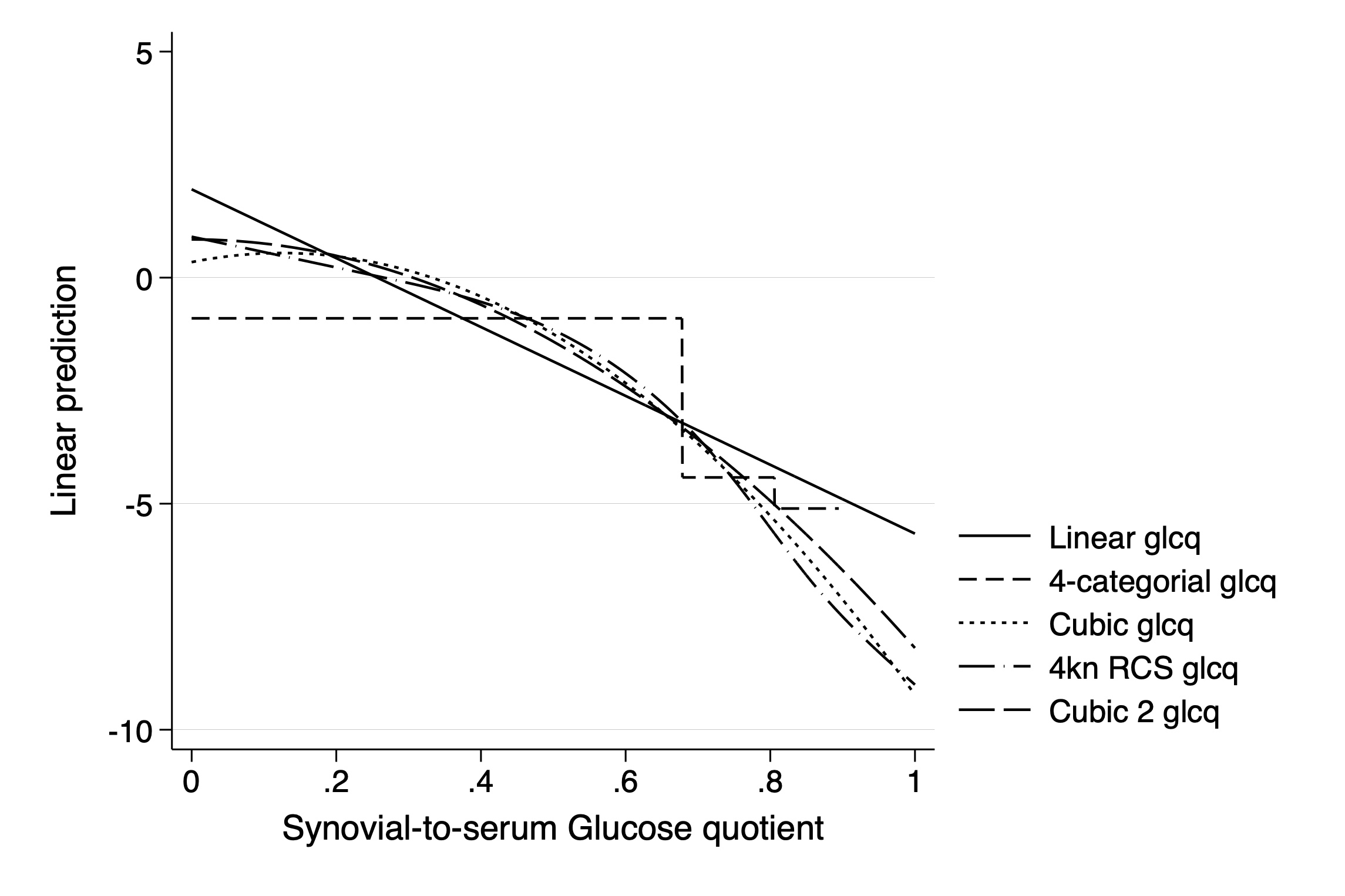
**

**eFigure S3.** Synovial-to-serum Glucose quotient relationship to the outcome

RCS; Restricted cubic spline

**
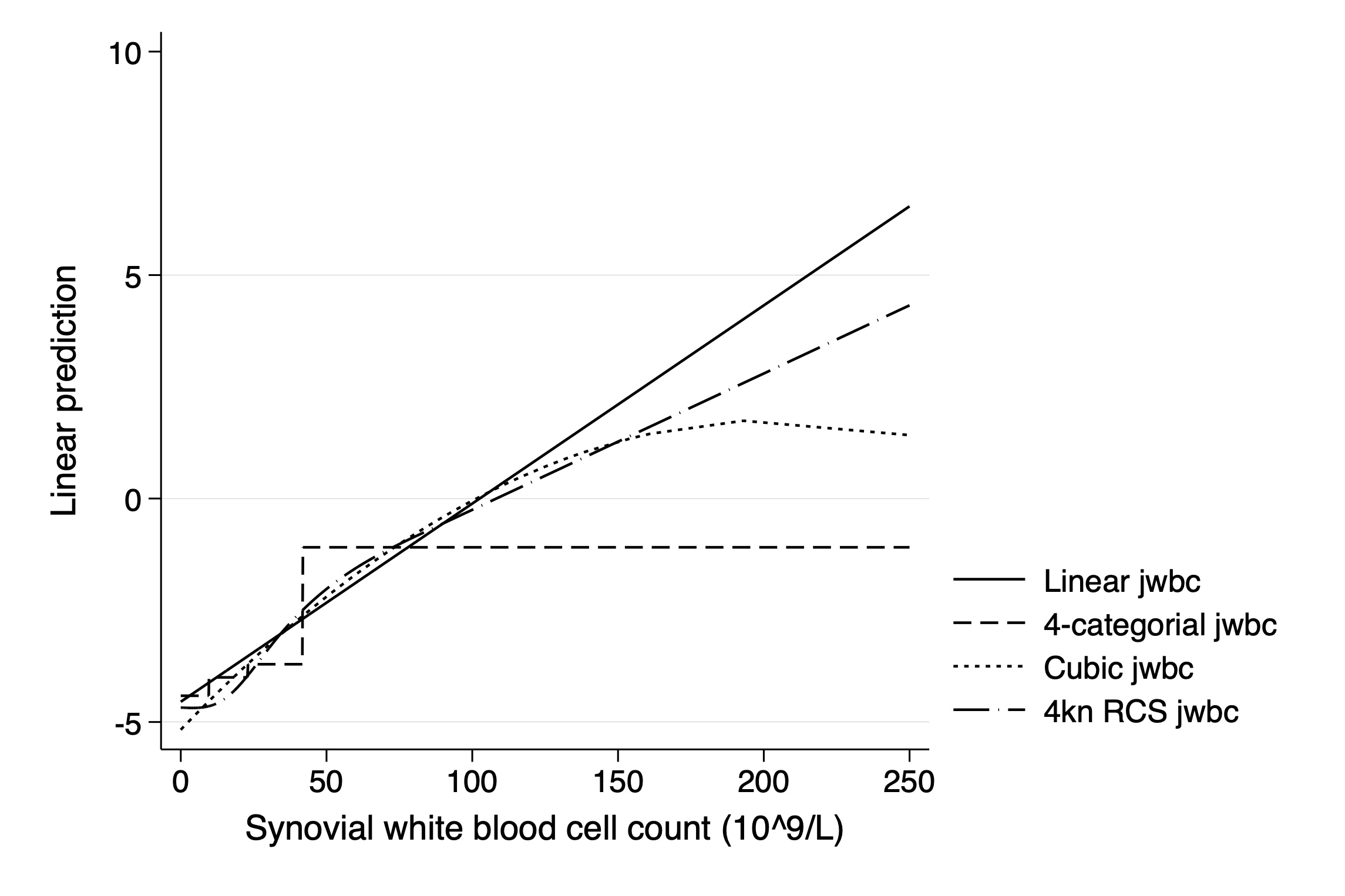
**

**eFigure S4.** Synovial white blood cell count relationship to the outcome

**
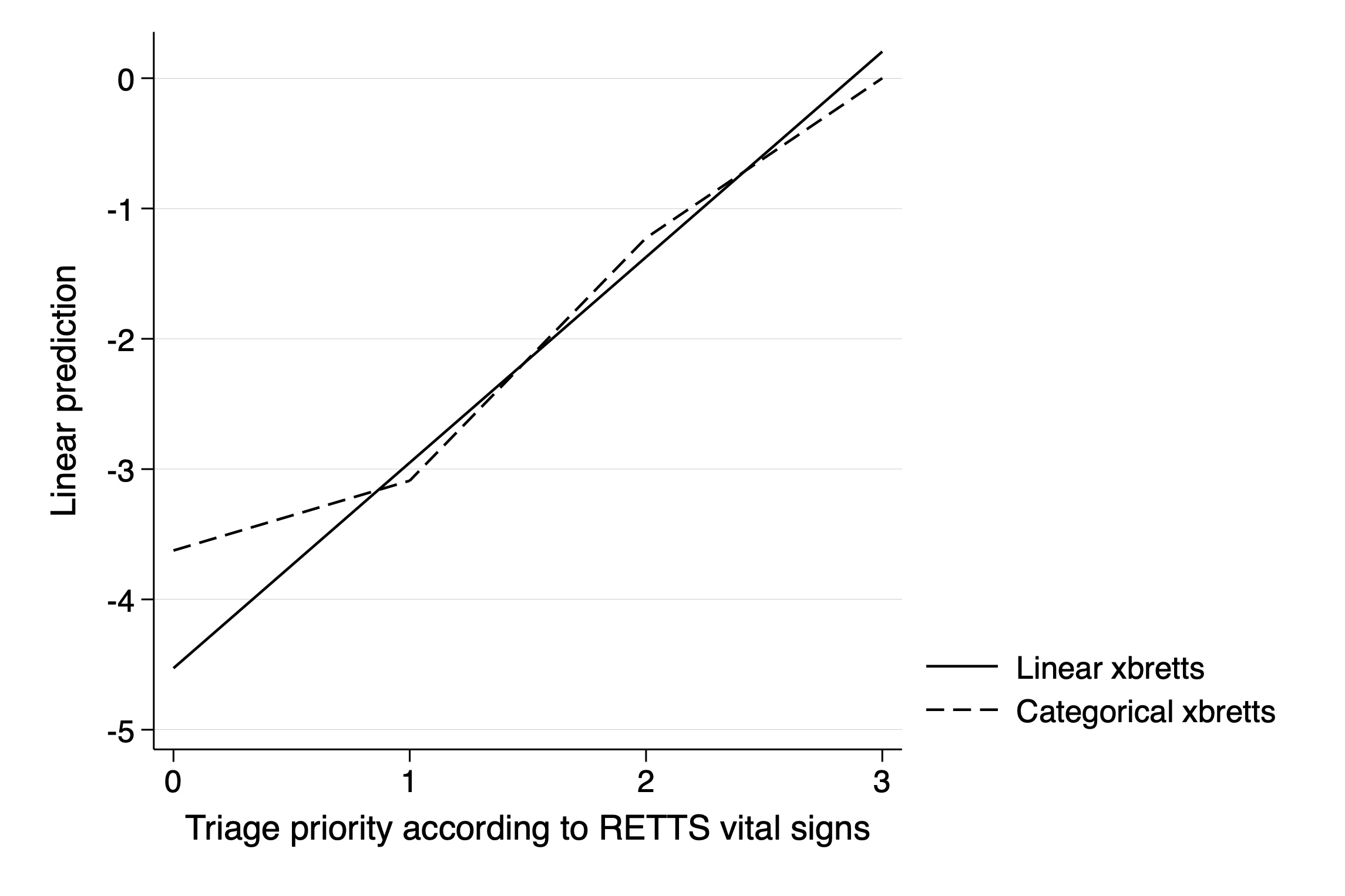
**

**eFigure S5.** Triage priority according to Rapid Emergency Triage and Treatment System vital signs relationship to the outcome

**eTable S3.** Univariate performance of pre-specified candidate predictors. All variables are analysed univariately on their original scale using logistic regression towards the primary outcome of septic arthritis.

| **Variable** | **Pseudo R^2^** | ***p* value** | **Odds ratio (95% CI)** | **Range** | **% missing** |
| --- | --- | --- | --- | --- | --- |
| *Primary candidates* | | | | | |
| Synovial-to-serum glucose quotient | 0.382 | <0.001 | 0.002 (0.000 to 0.008) | 0-250 | 29% |
| Synovial white blood cell count (10^9^/L) | 0.257 | <0.001 | 1.039 (1.029 to 1.050) | 0.00-1.00 | 12% |
| Abnormal synovial fluid by visual inspection * | 0.187 | <0.001 | 32.43 (7.784 to 135.1) | 0/1 | 8% |
| Serum C-reactive protein | 0.160 | <0.001 | 1.011 (1.008 to 1.014) | 0-463 | 5% |
| Skin redness over knee | 0.067 | <0.001 | 4.481 (2.283 to 8.088) | 0/1 | 3% |
| *Secondary candidates* | | | | | |
| Triage priority according to RETTS | 0.119 | <0.001 | 4.258 (2.686 to 6.748) | 0,1,2,3 | 14% |
| Temperature at ED | 0.065 | <0.001 | 2.189 (1.594 to 3.005) | 34.7-40.8 | 4% |
| Serum white cell count (10^9^/L) | 0.018 | 0.012 | 1.095 (1.020 to 1.176) | 2.4-28.8 | 21% |
| *Exploratory candidates* | | | | | |
| Joint range of motion | 0.062 | <0.001 | 2.346 (1.538 to 3.580) | 1,2,3,4 | 31% |
| Symptom duration (days) | 0.044 | 0.317 | 0.975 (0.927 to 1.025) | 0-730 | 6% |
| Rheumatoid arthritis | 0.013 | 0.017 | 3.157 (1.232 to 8.092) | 1/0 | 0% |
| Diabetes mellitus | 0.013 | 0.024 | 1.817 (1.08 to 3.05) | 0,1,2 | 0% |
| Charlson comorbidity index (points) | 0.009 | 0.071 | 1.096 (0.992 to 1.211) | 0-16 | 0% |
| Age (years) | 0.005 | 0.178 | 1.011 (0.995 to 1.028) | 18-97 | 0% |
| Female sex | 0.002 | 0.375 | 0.749 (0.340 to 1.417) | 1/0 | 0% |

*Range: “-“ means continuous variable, “,” means categorical variable and “/” is a dichotomous variable. % missing is a quotient of total n=668. RETTS; Rapid Emergency Triage and Treatment System (based on vital signs), ED; Emergency department. * Note that synovial fluid containing normal looking blood without pus or debris was not considered abnormal fluid.*

**Table S4.** Multivariable logistic regression parameters for the four variables in the final SAS prediction model.

| **Variable** | **Z value** | ***p* value** | **Odds ratio**  **(95% CI)** | **Bootstrap**  **adjusted**  **Odds ratio**  **(95% CI)** | |
| --- | --- | --- | --- | --- | --- |
| Synovial to serum glucose quotient  (Continuous variable from 0 to 1) | -6.83 | <0.001 | 0.0009  (0.0001 to 0.007) | 0.0009  (0.0001 to 0.002) | |
| Triage priority according to RETTS  (4 categories from lowest 0 to highest 3) | 4.81 | <0.001 | 5.6  (2.8 to 11.3) | 5.08  (2.62 to 9.87) |  |
| Synovial white blood cell count  (Continuous variable from 0 to 250) | 3.02 | 0.003 | 1.02  (1.01 to 1.03) | 1.02  (1.01 to 1.03) |  |
| Abnormal synovial fluid by visual inspection (Dichotomous variable) | 2.91 | 0.004 | 11.5  (2.2 to 59.8) | 10.0  (2.12 to 47.4) |  |

*Bootstrap adjusted OR is based on 0.943 shrinkage factor using 1000 replications bootstrap.*


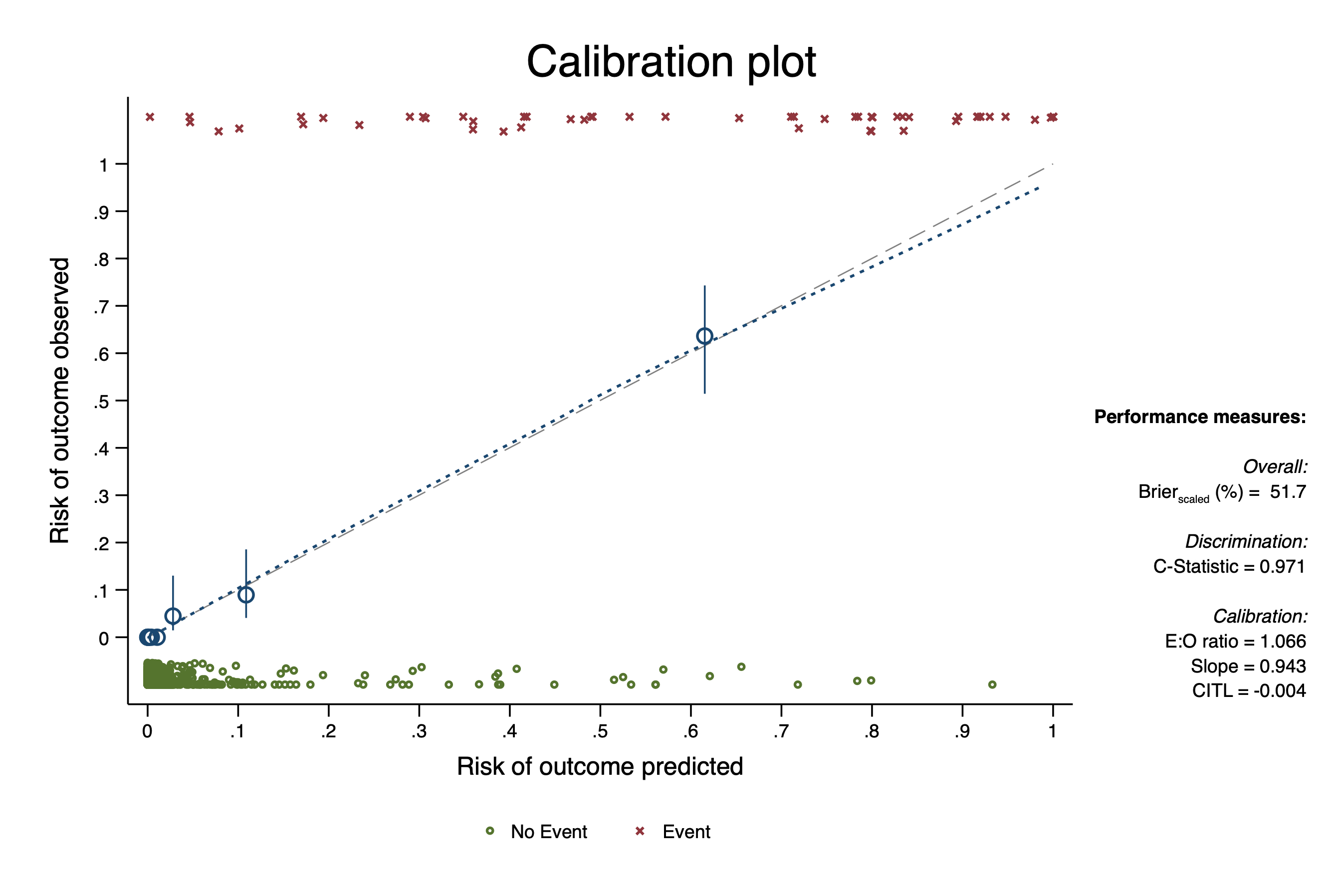


**eFigure S6.** Calibration plot for interval validation of prediction model performance. Based on 1000 replication bootstrap with expected-to-observed ratio, slope, calibration in the large (i.e., intercept), optimism adjusted C-statistic (i.e, area under receiver operating characteristics curve, AUROC) and scaled Brier score.

**eTable S5**. Nomogram table

| Synovial to serum glucose quotient | Synovial white blood cell count | Triage priority according to RETTS vital signs | Abnormal synovial fluid by visual inspection |
| --- | --- | --- | --- |
| 1 -> 0.0 points  .9 -> 1.0 points  .8 -> 2.0 points  .7 -> 3.0 points  .6 -> 4.0 points  .5 -> 5.0 points  .4 -> 6.0 points  .3 -> 7.0 points  .2 -> 8.0 points  .1 -> 9.0 points  0 -> 10.0 points | 0 -> 0.0 points  50 -> 1.5 points  100 -> 2.9 points  150 -> 4.4 points  200 -> 5.9 points  250 -> 7.4 points | 0 -> 0.0 points  1 -> 2.2 points  2 -> 4.3 points  3 -> 6.5 points | 0 -> 0.0 points  1 -> 3.1 points |

**eTable S6**. Antibiotics administered by clinicians according to medical chart review.

| **Name of Antibiotic** | **Outcome negative**  **(*n* = 617)** | **Outcome positive**  **(*n* = 51)** |
| --- | --- | --- |
| **Intravenous antibiotic** | | |
| Cloxacillin | 111 | 17 |
| Benzylpenicillin | 5 | 0 |
| Piperacillin-Tazobactam | 7 | 2 |
| Cefotaxime | 89 | 25 |
| Clindamycin | 0 | 1 |
| Meropenem | 0 | 1 |
| Vancomycin | 1 | 0 |
| Clindamycin + Gentamicin | 0 | 1 |
| Benzylpenicillin + Cloxacillin | 1 | 0 |
| *Total i.v. antibiotics* | *214 (35%)* | *47 (92%)* |
| **Oral antibiotics** | | |
| Flucloxacillin | 16 | 2 |
| Penicillin | 5 | 0 |
| Clindamycin | 3 | 0 |
| Ciprofloxacin | 1 | 0 |
| Doxycycline | 4 | 0 |
| Cefadroxil | 1 | 0 |
| *Total oral antibiotics* | *30 (5%)* | *2 (4%)* |
| **Total antibiotics** | 244 (40%) | 49 (96%) |
| *No empirical antibiotic* | 373 (60%) | 2 (4%) |


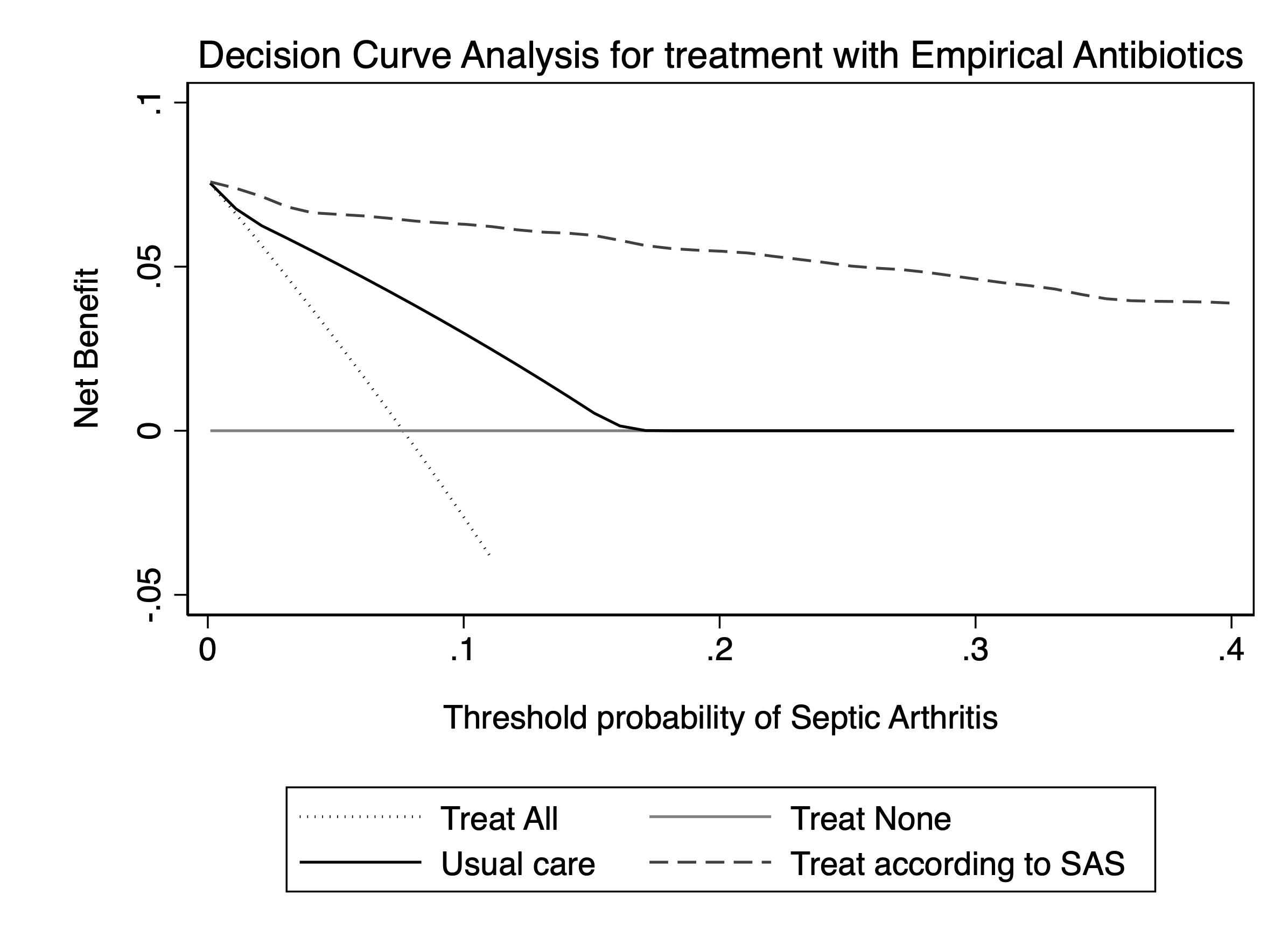


**eFigure S7.** Decision curve analysis graph with a comparison of Net Benefit for the four different strategies of treat all, treat none, usual care and the novel SAS prediction model across different treatment thresholds. Usual care is based medical chart review. Net benefit (NB) is defined as NB = benefit − (harm × exchange rate) where benefit is true positive rate (i.e., sensitivity) and harm is false positive rate and the exchange rate is based on how many patients one is willing to treat for every positive case (i.e., directly related to the treatment threshold).


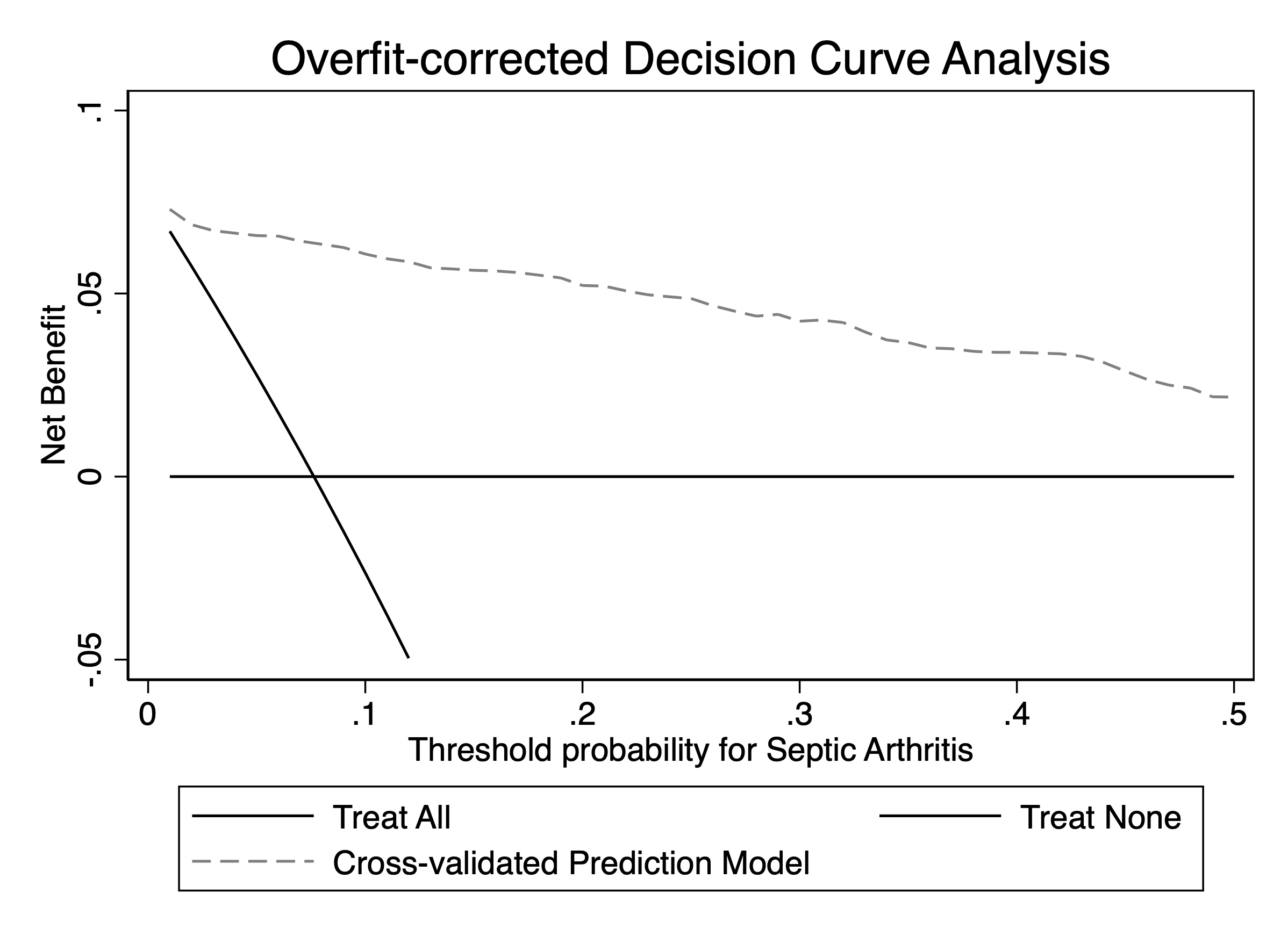


**eFigure S8.** Overfit-corrected decision curve analysis (DCA) graph.

**eTable S7.** Pre-specified microbiologial contaminants and pathogens

| **microorganism** | **pathogen_contaminant_SAS** |
| --- | --- |
| Abiotrophia species | Pathogen |
| Acinetobacter baumannii | Pathogen |
| Acinetobacter baumannii-gruppen | Pathogen |
| Acinetobacter species | Pathogen |
| Actinomyces species | Contaminant |
| Aerococcus sanguinicola | Contaminant |
| Aerococcus urinae | Contaminant |
| Aeromonas hydrophila | Pathogen |
| Alfastreptokock | Pathogen |
| Anaerob blandflora | Contaminant |
| Anaerococcus species | Pathogen |
| Bacillus cereus | Contaminant |
| Bacillus species | Contaminant |
| Bacteroides fragilis | Pathogen |
| Bacteroides fragilis gruppen | Pathogen |
| Bacteroides fragilis-gruppen och Porphyromonas species | Pathogen |
| Betahemolyserande streptokock grupp A (Strepto coccus pyogenes) | Pathogen |
| Betahemolyserande streptokock grupp A (Streptococcus pyogenes) | Pathogen |
| Betahemolyserande streptokock Grupp B (Strepto coccus agalactiae) | Pathogen |
| Betahemolyserande streptokock grupp C | Pathogen |
| Betahemolyserande streptokock grupp C eller G | Pathogen |
| Betahemolyserande streptokock grupp G | Pathogen |
| Betahemolyserande streptokocker (grupp C eller G) | Pathogen |
| Betahemolyserande streptokocker grupp G (Strep tococcus canis) | Pathogen |
| Borrelia afzelii | Pathogen |
| Borrelia burgdorferi | Pathogen |
| Borrelia species | Pathogen |
| Brucella melitensis | Pathogen |
| Campylobacter jejuni | Pathogen |
| Candida albicans | Pathogen |
| Candida parapsilosis | Pathogen |
| Candida tropicalis | Pathogen |
| Capnocytophaga canimorsus | Pathogen |
| Citrobacter freundii | Pathogen |
| Citrobacter koseri | Pathogen |
| Citrobacter species | Pathogen |
| Clostridium perfringens | Pathogen |
| Corynebacterium amycolatum | Contaminant |
| Corynebacterium species | Contaminant |
| Corynebacterium striatum | Contaminant |
| Corynebacterium striatum och Enterococcus faec alis | Pathogen |
| Cryptococcus neoformans | Pathogen |
| Cupriavidus species och Pandoraea species | Contaminant |
| Cutibacterium (Propionibacterium) acnes | Contaminant |
| Cutibacterium (Propionibacterium) granulosum | Contaminant |
| Cutibacterium (Propionibacterium) species | Contaminant |
| Enterobacter aerogenes | Pathogen |
| Enterobacter cloacae | Pathogen |
| Enterobacter species | Pathogen |
| Enterococcus faecalis | Pathogen |
| Enterococcus faecium | Pathogen |
| Enterococcus gallinarum | Pathogen |
| Enterococcus hirae | Pathogen |
| Enterococcus species | Pathogen |
| Enterococcus species och Staphylococcus specie s | Contaminant |
| Escherichia coli | Pathogen |
| Esherichia coli | Pathogen |
| Finegoldia magna | Pathogen |
| Fusobacterium nucleatum och Prevotella oris | Pathogen |
| Fusobacterium species | Pathogen |
| Fusobacterium species och Prevotella species | Pathogen |
| För många olika arter för att kunna särskilja | NULL |
| Gemella species | Pathogen |
| Gramnegativ blandflora | Pathogen |
| Gramnegativ stav | Pathogen |
| Gramnegativ stav, ej Enterobacteriaceae | Pathogen |
| Gramnegativ stav, Enterobacteriaceae | Pathogen |
| Gramnegativ stav, odlingskrävande art | Pathogen |
| Grampositiv kock | Pathogen |
| Grampositiv kock, anaerob | Pathogen |
| Grampositiv kock, misstänkt enterokock eller streptokock | Pathogen |
| Grampositiv kock, misstänkt stafylokock | Contaminant |
| Grampositiv stav | Pathogen |
| Grampositiv stav, anaerob | Pathogen |
| Granulicatella adiacens | Pathogen |
| Granulicatella species | Pathogen |
| Grupp B streptokock (Streptococcus agalactiae) | Pathogen |
| Haemophilus influenzae | Pathogen |
| Haemophilus parainfluenzae | Pathogen |
| Hortaea werneckii | Pathogen |
| hudflora | Contaminant |
| Jästsvamp | Contaminant |
| Kingella kingae | Pathogen |
| Klebsiella (Enterobacter) aerogenes | Pathogen |
| Klebsiella eller Enterobacter | Pathogen |
| Klebsiella eller Enterobacter samt Staphylococ cus spp | Pathogen |
| Klebsiella oxytoca | Pathogen |
| Klebsiella pneumoniae | Pathogen |
| KNS (koagulasnegativ Staphylococcus) | Contaminant |
| Koagulas-negativ stafylokock (KNS) | Contaminant |
| Koagulasnegativa stafylokocker | Contaminant |
| Kocuria rosea | Contaminant |
| Lactococcus species | Contaminant |
| Legionella bozemanii | Pathogen |
| Micrococcus luteus | Contaminant |
| Micrococcus species | Contaminant |
| Moraxella (Branhamella) catarrhalis | Pathogen |
| Moraxella species | Pathogen |
| Morganella morganii | Pathogen |
| Mycoplasma hominis | Pathogen |
| Neisseria gonorrhoeae | Pathogen |
| Neisseria meningitidis | Pathogen |
| Neisseria species | Pathogen |
| Neisseria species, apatogen art | Contaminant |
| Paracoccus species | Contaminant |
| Parastreptomyces abscessus | Contaminant |
| Parvimonas micra | Contaminant |
| Parvimonas micra och Peptostreptococcus specie s | Contaminant |
| Pasteurella multocida | Pathogen |
| Peptoniphilus species | Contaminant |
| Peptostreptococcus anaerobius | Contaminant |
| Prevotella species | Pathogen |
| Propionibacterium acnes | Contaminant |
| Propionibacterium granulosum | Contaminant |
| Propionibacterium species | Contaminant |
| Proteus mirabilis | Pathogen |
| Proteus species | Pathogen |
| Proteus vulgaris gruppen | Pathogen |
| Pseudomonas aeruginosa | Pathogen |
| Pseudomonas species | Pathogen |
| Pseudomonas stutzeri | Pathogen |
| Raoultella planticola | Pathogen |
| Rothia mucilaginosa | Contaminant |
| Rothia species | Contaminant |
| Serratia marcescens | Pathogen |
| Serratia marcescens och Streptococcus pyogenes (grupp A strept.) | Pathogen |
| Staphylococcus aureus | Pathogen |
| Staphylococcus capitis | Contaminant |
| Staphylococcus caprae | Contaminant |
| Staphylococcus epidermidis | Contaminant |
| Staphylococcus epidermis | Contaminant |
| Staphylococcus lugdunensis | Pathogen |
| Staphylococcus saccharolyticus | Contaminant |
| Staphylococcus species | Contaminant |
| Streptococcus agalactiae (Betahemolyserande st reptokock gr B) | Pathogen |
| Streptococcus anginosus (milleri) gruppen | Contaminant |
| Streptococcus bovis gruppen | Contaminant |
| Streptococcus canis (tillhör grupp G streptoko cker) | Pathogen |
| Streptococcus dysgalactiae | Pathogen |
| Streptococcus dysgalactiae (Betahemolyserande streptokock gr G/C) | Pathogen |
| Streptococcus dysgalactiae subsp dysgalactiae | Pathogen |
| Streptococcus dysgalactiae subsp euisimilis | Pathogen |
| Streptococcus mitis gruppen | Pathogen |
| Streptococcus mitis/sanguinis gruppen | Pathogen |
| Streptococcus mitisgruppen(alfahemolytiska str eptokocker) | Pathogen |
| Streptococcus mutans | Pathogen |
| Streptococcus pneumoniae (Pneumokock) | Pathogen |
| Streptococcus salivarius gruppen | Pathogen |
| Streptococcus sanguinis gruppen | Pathogen |
| Streptococcus species | Pathogen |
| Streptomyces species | Pathogen |
| Trådsvamp | Contaminant |
| Ureaplasma species | Contaminant |
| Varicella zoster | Contaminant |
| Veillonella species | Contaminant |

**eReferences**

1. Margaretten ME, Kohlwes J, Moore D, Bent S. Does this adult patient have septic arthritis? JAMA. 2007;297(13):1478-88.

2. Carpenter CR, Schuur JD, Everett WW, Pines JM. Evidence-based diagnostics: adult septic arthritis. Acad Emerg Med. 2011;18(8):781-96.

3. Mellhammar L, Linder A, Tverring J, Christensson B, Boyd JH, Åkesson P, et al. Scores for sepsis detection and risk stratification - construction of a novel score using a statistical approach and validation of RETTS. PLoS One. 2020;15(2):e0229210.

4. Vickers AJ, Van Calster B, Steyerberg EW. Net benefit approaches to the evaluation of prediction models, molecular markers, and diagnostic tests. BMJ. 2016;352:i6.
