# Supplementary material for: Septic arthritis score (SAS) – a novel clinical prediction model for the probability of septic arthritis in the adult native knee
Source: BMC Infect Dis. 2025 Jul 18;25:926. doi: 10.1186/s12879-025-11306-6 (PMC12275251; doi:10.1186/s12879-025-11306-6)
Supplement: Supplementary file 3 — Supplementary Material 3. [file 12879_2025_11306_MOESM3_ESM.docx]

/// Stata do-file final for the SAS manuscript ///

* Title: Septic Arthritis Score (SAS) – A novel clinical prediction model for the probability of septic arthritis in the adult native knee

* Version 1.0, Date: 13mar2024

* Author: Jonas Tverring

* Stata/MP 18.0

/// 1. Explore pre-specified candidate predictors

* Primary candidates

logistic outc jwbc, nolog

logistic outc glcq, nolog

logistic outc jlooks, nolog

logistic outc scrp, nolog

logistic outc jredness, nolog

* Glcq and jwbc are clearly strongest as suspected. Jlooks and scrp are similar and jredness weaker.

*Secondary candidates

logistic outc retts, nolog

logistic outc temp, nolog

logistic outc swbc, nolog

* RETTS (vital signs) is stronger than temp as suspected and it's reasonable to keep vital signs to differentiate infection. Swbc is weak as often.

* Exploratory candidates

logistic outc jpain_palp, nolog

logistic outc jpain_bearweight , nolog

logistic outc duration, nolog

logistic outc age, nolog

logistic outc female, nolog

logistic outc charlson, nolog

logistic outc jrom, nolog

logistic outc ra, nolog

logistic outc diabetes, nolog

* Most of these are weak as suspected. Jrom is the exception which is at level with jredness.

* Let's look at jlooks and retts to see if they add value to jwbc and jglcq

quietly logistic outcome glcq jwbc scrp jlooks

est store G

quietly logistic outcome glcq jwbc scrp if jlooks!=.

est store H

lrtest G H

quietly logistic outcome glcq jwbc scrp jlooks

est store Q

quietly logistic outcome glcq jwbc jlooks if scrp!=.

est store R

lrtest Q R

*jLOOKs and sCRP but add value, but CRP only barely

quietly logistic outcome glcq jwbc jlooks scrp retts

est store I

quietly logistic outcome glcq jwbc jlooks scrp if retts!=.

est store J

lrtest I J

* RETTS adds value also when jlooks and CRP has been added, however CRP looks weak in the whole model, let's see if it adds value with the other 4 variables (and the same for jlooks to be fair)

logistic outcome glcq jwbc jlooks scrp retts

est store S

quietly logistic outcome glcq jwbc jlooks retts if scrp!=.

est store T

lrtest S T

quietly logistic outcome glcq jwbc jlooks scrp retts

est store X

quietly logistic outcome glcq jwbc retts scrp if jlooks!=.

est store Y

lrtest X Y

* jLOOKS still adds value but CRP does not.

* CRP, Jrom and Jredness could be discussed as reasonable candidates that are also fairly strong univariately.

* Let's look at these variables multivariably with the variables we chose to incldude with certainty.

quietly logistic outcome glcq jwbc retts jlooks jredness

est store A

quietly logistic outcome glcq jwbc retts jlooks if jredness!=.

est store B

lrtest A B

quietly logistic outcome glcq jwbc retts jlooks jrom

est store E

quietly logistic outcome glcq jwbc retts jlooks if jrom!=.

est store F

lrtest E F

quietly logistic outcome glcq jwbc retts jlooks temp

est store K

quietly logistic outcome glcq jwbc retts jlooks if temp!=.

est store L

lrtest K L

* None of them adds to the likelihood ratio chi(2) test.

drop _est*

/// 2. Variable tranformations (investigating non-linearity of potential predictors)

egen iglcq=cut(glcq), group(4)

egen ijwbc=cut(jwbc), group(4)

gen jwbcsq=jwbc^2

gen glcqsq=glcq^2

mkspline2 jwbcs = jwbc, cubic nknots(4)

mkspline2 glcqs = glcq, cubic nknots(4)

* Categorical variables (RETTS)

* Linear

logistic outcome retts

predict xbretts, xb

label variable xbretts "Linear xbretts"

est store A

* Categorical

logistic outcome i.retts

predict ixbretts, xb

label variable ixbretts "Categorical xbretts"

est store B

* graph

tw (line xbretts ixbretts retts, sort) , ytitle(Linear prediction) xtitle(Vital Signs (RETTS)) name(xbretts, replace) graphregion(lcolor(white))

lrtest A B

drop _est*

drop xb*

* i.e. linear is as good as categorical (p=0.76) and graph looks similar.

* Cont. variables (glcq)

* Linear:

logistic outcome glcq

predict xbglcq, xb

label variable xbglcq "Linear glcq"

est store A

* Categorical:

logistic outcome i.iglcq

predict xbiglcq, xb

label variable xbiglcq "4-categorial glcq"

* Cubic:

logistic outcome glcq glcqsq

predict xbglcqsq, xb

label variable xbglcqsq "Cubic glcq"

est store B

* 3 Knot Restricted cubic spline (4 kn RCS):

logistic outcome glcqs1 glcqs2 glcqs3

predict xbglcqs, xb

label variable xbglcqs "4kn RCS glcq"

est store C

lrtest A B

lrtest A C

* Graph:

tw (line xbglcq xbiglcq xbglcqsq xbglcqs glcq, sort) , ytitle(Linear prediction) xtitle(Joint-Serum Glucose Ratio) name(xbglcq, replace) graphregion(lcolor(white))

drop _est*

drop xb*

* Both spline and cubic glcq are a little better numerically than linear glcq and the graph shows that it's account for by a non-linear decrease in risk when glq approaches 1

* Both it's no significant difference in likelihood ratio and may be a result of over-fitting to the data so I'll stick with linear, also for the sake of saving degrees of freedom.

* Joint leucyte count

* Linear:

logistic outcome jwbc

predict xbjwbc, xb

label variable xbjwbc "Linear jwbc"

est store A

* Categorical:

logistic outcome i.ijwbc

predict xbijwbc, xb

label variable xbijwbc "4-categorial jwbc"

* Cubic:

logistic outcome jwbc jwbcsq

predict xbjwbcsq, xb

label variable xbjwbcsq "Cubic jwbc"

* 3 Knot Restricted cubic spline (3 kn RCS):

logistic outcome jwbcs1 jwbcs2 jwbcs3

predict xbjwbcs, xb

label variable xbjwbcs "4kn RCS jwbc"

est store B

lrtest A B

* Graph:

tw (line xbjwbc xbijwbc xbjwbcsq xbjwbcs jwbc, sort) , ytitle(Linear prediction) xtitle("Joint Leucocyte count") name(xbjwbc, replace) graphregion(lcolor(white))

drop _est*

drop xb*

* Linear is as good as spline numiercally and graphically.

/// 3. Final prediction model specification

logistic outcome glcq jwbc retts jlooks

est store cc

/// 4. Explore missing data

misstable sum outcome glcq jwbc retts jlooks

* So a large degree of missing particularly in our most influential variable, glcq 29%. 12% in jwbc, 14% in retts and 8% in jlooks. We definately need to consider imputation.

misstable patterns outcome glcq jwbc retts jlooks

* 15% are missing only glcq, and 8% only retts and 7% glcq and jwbc, 5% only jlooks.

misstable sum jglc sglc

* Missing glcq if mostly due to missing serum glucose and that can be consided random.

* In knee taps swbc and culture are normally prioritized over jglc so there may be a non-random component for the amount of fluid relative to missingness, but we do not have this data and it could also be that the physician simply forgot to order it. The same goes for jwbc, may be related to amount of fluid.

* Most all patients should've gone through RETTS, so this is reasonably missing at random.

* And being a retrospective study jlooks may just have been left out of the chart.

corr glcq jwbc retts jlooks outcome female age prevab2v empab ra diabetes prevop admdays bdate

* looks like all missing variables have some fairly strong correlations to candidate nonmissing variables

* Impute missing data

mi set mlong

mi register imputed glcq jwbc retts jlooks

mi impute chained (pmm, knn(10)) glcq jwbc retts jlooks = outcome female age prevab2v empab ra clf diabetes prevop admdays bdate, add(40) rseed(1222) replace

* MI diagnostics

quietly mi estimate: logistic outcome glcq jwbc retts jlooks

how_many_imputations

* Number of imputations is adequate (37 needed, 40 imputed)

midiagplots glcq, combine

midiagplots jwbc, combine

midiagplots retts, combine

midiagplots jlooks, combine

* MI diagnostic plots look good

/// 6. Define MI prediction model and run model diagnostics

mi estimate, eform saving(miest, replace): logit outcome glcq jwbc retts jlooks

est store mi

* Saving MI model

* Generate MI-dervied probabilites for a positive outcome from the MI model.

mi predict xb_mi using miest

mi xeq: generate phat = invlogit(xb_mi)

* Model calibration

calibrationbelt outcome phat, devel(internal) name(calbetmi, replace) ci(95)

* Test of miscalibration not significant and plot looks reasonable

pmcalplot phat outcome , name(calplotmi2, replace) p(4) ci

* calibration in the large 0.104, slope 1.152, expected:observed 0.953 and AUC 0.97. Looks good.

* Collapse MI data to allow for classification and nomolog.

collapse (median) id nr outcome retts jlooks empab adeqab guidelineab ivab dtadm prevab2v empiricab01 cultbact01 bcultbact01 phat admdays daytodead bdate otherdiag01 (mean) time_to_ab glcq jwbc scrp _mi_miss _mi_m, by(_mi_id)

gen _miss=_mi_m

replace _mi_m=0

mi extract 0, clear

replace retts=round(retts)

replace jlooks=round(jlooks)

* Full prediction model on collapsed MI data

logistic outcome glcq jwbc retts jlooks

est store coll

predict pr, pr

* Explore differences in coefficients between complete case data, MI data and collapsed MI data. Some difference in glcq and small diff in jlooks

coefplot mi cc coll, drop(_cons) eform xline(1) xscale(log) xlabel(0.001 0.01 0.1 0 10 100) xtitle(Odds ratio and 95% CI) name(coefplotmeanmedian, replace)

* Model diagnostics on collapsed data

collin outcome glcq jwbc retts jlooks

* no collinearity. VIF below 2 in all. Condition number below 15 (14.8)

logistic outcome glcq jwbc retts jlooks

estat vce

* Influential observations

logistic outcome glcq jwbc retts jlooks

ldfbeta

sum DF*

* all below 0,4 but some above 0,2.

list outcome glcq jwbc retts jlooks scrp pr DFjlooks if DFjlooks>0.2 & DFjlooks!=. | DFjlooks<-0.2 & DFjlooks!=.

* These are all influential because synovial fluid looked normal while most other factors did not. Not unrealistic I would say.

list outcome glcq jwbc retts jlooks scrp pr DFglcq if DFglcq>0.2 & DFglcq!=. | DFglcq<-0.2 & DFglcq!=.

* we have four patient with very low glcq that leads to a high risk, but then ends up having a negative outcome and then 2 patients with high glcq and low risk despite a positive outcome. Also not unreasonable.

* Screening for some pre-defined clinically relevant interactions

logistic outcome c.glcq##(c.jwbc retts jlooks)

testparm c.glcq##(c.jwbc i.retts i.jlooks)

* None found

//// 5 Model performance - calibration, fit, discrimination

logistic outcome glcq jwbc retts jlooks

lroc, nograph

roctab outc pr

* Raw ROC area is 0.9713 with 95% CI: 0.95756 to 0.98507

cvauroc outcome glcq jwbc retts jlooks scrp, kfold(20) seed(1234) probit

* Optimism corrected ROC area is cvMean AUC: 0.9700 with Bootstrap bias corrected 95%CI: | 0.9446, 0.9811

overfit: logistic outcome glcq jwbc retts jlooks

* Overfitting is ~ 3.10%

quietly logistic outcome glcq jwbc retts jlooks

bsvalidation, rseed(1224) graph adjust(bootstrap) reps(500)

* Calibration is good, CITL/Intercept 0.10, E:O 1.025 and slope 0.943

/// 6. Create nomogram and investigate classification optimal cut-offs

logistic outcome glcq jwbc retts jlooks

nomolog, vli1(jlooks,0,1,1,0) vli2(retts,0,3,1,0) vli3(jwbc,0,250,50,0) vli4(glcq,0,1,0.1,1) varlabdescr title("Septic Arthitis Score (SAS) - Probability for Culture Positivity") divtable

/// 7. Estimate optimal cut-offs and clinical correlate

* Clinician performance according to usual care in dataset

tab outc guidelineab

* clinicians provided s.a active iv ab to 54 out of 58 patients

tab outc empab

* and 258 patients with neg outcome got oral or iv ab

logistic outc empab

estat class, cutoff(0.10)

* corresponding to 5,3 ab doses per positive case or 93 sens, 60% specificity and 63% correct classification

* we can achieve the same 93% sens by using SAS if we have 10% treatment threshold

quietly logistic outcome glcq jwbc retts jlooks

logistic outcome glcq jwbc retts jlooks

estat class, cutoff(0.10)

* Or we achieve 100% sens at 2% treatment threshold.

quietly logistic outcome glcq jwbc retts jlooks

estat class, cutoff(.02)

* SAS Positive Likelihood ratio at 10% threshold probability (LR+ = Sensitivity / (1 – specificity))

di 0.92/(1-0.92)

* LR+ 11.5

* SAS Negative Likelihood ratio at 10% threshold probability (LR- = (1 – sensitivity) / (specificity))

di (1-0.92)/0.92

* LR- 0.087

* SAS Positive Likelihood ratio at 2% threshold probability (LR+ = Sensitivity / (1 – specificity))

di 1/(1-0.79)

* LR+ 4.8

* SAS Negative Likelihood ratio at 2% threshold probability (LR- = (1 – sensitivity) / (specificity))

di (1-1)/0.79

* LR- 0

/// 8. Decision curve analyses and Net benefit (NB)

* DCA with clinicians decision vs using SAS

dca outcome empab pr, probability(no yes) smooth xstop(0.4) lcolor(black gs8 black) lpattern(dot solid solid dash) title("Decision Curve Analysis for treatment with Empirical Antibiotics", size(4) color(black)) scheme(s1mono) saving(SASdca.dta, replace) legend(label(1 "Treat All") label(2 "Treat None") label(3 "Usual care") label(4 "Treat according to SAS") rows(2)) xtitle("Threshold probability of Septic Arthiris") interventionper(10)

* DCA looking at guidelineab instead

dca outcome empab guidelineab pr, probability(yes yes yes) smooth xstop(0.4) lcolor(gs8 black black gs8 black) lpattern(dot dot solid solid dash) title("Decision Curve Analysis for treatment with Empirical Antibiotics", size(4) color(black)) scheme(s1mono) saving(SASdca.dta, replace) legend(label(1 "Treat All") label(2 "Treat None") label(3 "Usual care - any AB") label(4 "Usual case - i.v. AB") label(5 "Treat according to SAS") rows(3) size(3)) xtitle("Threshold probability of Septic Arthiris")

* Interventions avoided

dca outc pr, prob(yes) intervention xstart(0.01) xstop(0.35) scheme(s1mono) legend(rows(2)) title("Net reduction in empirical antibiotics per 100 patients") ytitle("Number of Antibiotic doses") xtitle("Threshold probability for treatment") legend(label(1 "Usual care") label(2 "Septic Arthritis Score"))

/// 9. Making a graph of patients missed versus antibiotic doses saved per threshold

gen th=_n/100

gen sasmiss=.

gen absave=.

forval i = 1(1)35 {

quietly logistic outcome glcq jwbc retts jlooks

quietly estat class, cutoff(`i'/100)

di ((r(P_n1)*60)/100) in `i'

}

forval i = 0.00(0.01)0.35 {

quietly logistic outcome glcq jwbc retts jlooks

quietly estat class, cutoff(`i')

di ((r(P_0p))/100)*((r(P_p1)*0.60)+(r(P_p0)*6.49))

}

tw (connected sasmiss th) (connected absave th) in 2/35, xlabel(0.025(0.025)0.35) ylabel(0 50 100 150 200) scheme(s1mono) legend(label(1 "SA patients not receiving empirical antibiotics (False negative)") label(2 "Non-SA patients receiving empirical antibiotics (False positive)") rows(2) size(3)) ytitle("Number of patients") xtitle("Threshold probability for treatment", size(3)) title("Trade-off from using SAS across different SA risk thresholds", size(3)) xline(0.005)

/// 10. Net benefit DCA overfit correction

forvalues i=1(1)2 {

local prediction1 = "model"

* Create variables to later store probabilities from each prediction model

quietly g `prediction1'=.

* Create a variable to be used to `randomize' the patients.

quietly g u = uniform()

* Sort by the event to ensure equal number of patients with the event are in each

* group

sort outcome u

* Assign each patient into one of ten groups

g group = mod(_n, 10) + 1

* Loop through to run through for each of the ten groups

forvalues j=1(1)10 {

* First for the "base" model:

* Fit the model excluding the jth group.

quietly logistic outcome glcq jwbc retts jlooks if group!=`j'

* Predict the probability of the jth group.

quietly predict ptemp if group==`j'

* Store the predicted probabilities of the jth group (that was not used in

* creating the model) into the variable previously created

quietly replace `prediction1' = ptemp if group==`j'

* Dropping the temporary variable that held predicted probabilities for all

* patients

drop ptemp

}

* Creating a temporary file to store the results of each of the iterations of our

* decision curve for the multiple the 10 fold cross validation

* This step may omitted if the optional forvalues loop was excluded.

tempfile dca`i'

* Run decision curve, and save the results to the tempfile.

* For those excluding the optional multiple cross validation, this decision curve

* (to be seen by excluding "nograph") and the results (saved under the name of your

* choosing) would be the decision curve corrected for overfit.

quietly dca outcome `prediction1', xstop(.5) nograph ///

saving("`dca`i''")

drop u group `prediction1'

} // This closing bracket ends the initial loop for the multiple cross validation.

* It is also necessary for those who avoided the multiple cross validation

* by changing the value of the forvalues loop from 200 to 1*/

* The following is only used for the multiple 10 fold cross validations.

use "`dca1'", clear

forvalues i=2(1)2 {

* Append all values of the multiple cross validations into the first file

append using "`dca`i''"

}

* Calculate the average net benefit across all iterations of the multiple

* cross validation

collapse all none model model_i, by(threshold)

save "Cross Validation DCA Output.dta", replace

* Labeling the variables so that the legend will have the proper labels

label var all "Treat All"

label var none "Treat None"

label var model "Cross-validated Prediction Model"

* Plotting the figure of all the net benefits.

twoway (line all threshold if all>-0.05, sort lcolor(black)) || (line none model threshold, lpattern(solid dash) lcolor(black) sort) , scheme(s1mono) xtitle("Threshold probability for Septic Arthritis") ytitle("Net Benefit") title("Overfit-corrected Decision Curve Analysis")

/// 11. Sensitivity analyses

* Without previous antibiotics

logistic outcome glcq jwbc retts jlooks if prevab2v==0

lroc, nograph

* pseudo R2 0.58 and AUROC 0.971 in n=611

* Without high Dfbeta *

logistic outcome glcq jwbc retts jlooks

ldfbeta

logistic outcome glcq jwbc retts jlooks if DFjlooks<0.2 & DFjlooks>-0.2 & DFjlooks!=. & DFglcq<0.2 & DFglcq>-0.2 & DFglcq!=.

lroc, nograph

* Pseudo R2 0.70 and AUROC 0.985 in n=700
